# A DL-4- and TNFα-based culture system to generate high numbers of nonmodified or genetically modified immunotherapeutic human T-lymphoid progenitors

**DOI:** 10.1038/s41423-021-00706-8

**Published:** 2021-06-11

**Authors:** Ranjita Devi Moirangthem, Kuiying Ma, Sabrina Lizot, Anne Cordesse, Juliette Olivré, Corinne de Chappedelaine, Akshay Joshi, Agata Cieslak, John Tchen, Nicolas Cagnard, Vahid Asnafi, Antonio Rausell, Laura Simons, Julien Zuber, Tom Taghon, Frank J. T. Staal, Françoise Pflumio, Emmanuelle Six, Marina Cavazzana, Chantal Lagresle-Peyrou, Tayebeh Soheili, Isabelle André

**Affiliations:** 1 Université de Paris, Imagine Institute, Laboratory of Human Lymphohematopoiesis, INSERM UMR 1163, Paris, France; 2 Laboratory of Onco-Hematology, AP-HP, Hôpital Necker-Enfants Malades., Paris, France; 3 Université de Paris, Institut Necker-Enfants Malades (INEM), INSERM UMR 1151, Paris, France; 4 Plateforme Bio-informatique, Université Paris Descartes, Structure Fédérative de Recherche Necker, INSERM US24/CNRS UMS 3633, Paris, France; 5 Université de Paris, Imagine Institute, Laboratory of Clinical Bioinformatics, INSERM UMR 1163, Paris, France; 6 Department of Biotherapy Clinical Investigation Center, AP-HP, Hôpital Necker-Enfants Malades, Paris, France; 7 Department of Adult Kidney Transplantation, AP-HP, Hôpital Necker, Paris, France; 8 Cancer Research Institute Ghent (CRIG), Ghent, Belgium; 9 Department of Diagnostic Sciences, Faculty of Medicine and Health Sciences, Ghent University, Ghent, Belgium; 10 Department of Immunohematology & Blood Transfusion, Leiden University Medical Center, Leiden, the Netherlands; 11 Team Niche and Cancer in Hematopoiesis, Université de Paris and Université Paris-Saclay, INSERM, iRCM/IBFJ CEA, UMR Stabilité Génétique Cellules Souches et Radiations, Fontenay-aux-Roses, France

**Keywords:** Human T-lymphoid progenitor, Tumor necrosis factor alpha, Delta-like ligand 4, Hematopoietic stem and progenitor cells, Mobilized peripheral blood, Transplant immunology, Lymphopoiesis

## Abstract

Several obstacles to the production, expansion and genetic modification of immunotherapeutic T cells in vitro have restricted the widespread use of T-cell immunotherapy. In the context of HSCT, delayed naïve T-cell recovery contributes to poor outcomes. A novel approach to overcome the major limitations of both T-cell immunotherapy and HSCT would be to transplant human T-lymphoid progenitors (HTLPs), allowing reconstitution of a fully functional naïve T-cell pool in the patient thymus. However, it is challenging to produce HTLPs in the high numbers required to meet clinical needs. Here, we found that adding tumor necrosis factor alpha (TNFα) to a DL-4-based culture system led to the generation of a large number of nonmodified or genetically modified HTLPs possessing highly efficient in vitro and in vivo T-cell potential from either CB HSPCs or mPB HSPCs through accelerated T-cell differentiation and enhanced HTLP cell cycling and survival. This study provides a clinically suitable cell culture platform to generate high numbers of clinically potent nonmodified or genetically modified HTLPs for accelerating immune recovery after HSCT and for T-cell-based immunotherapy (including CAR T-cell therapy).

## Introduction

T cells are the armed wing of the immune system that protect against viruses and cancer cells. Genetically engineered T cells (and particularly specific chimeric antigen receptor (CAR)-expressing T cells), mostly adult, now constitute an essential tool for T-cell immunotherapy (e.g., the treatment of lymphoma and HIV infection).^1,2^ Beyond effector T cells, regulatory T cells (Tregs, including CAR Tregs) have also been used to induce immune tolerance in the contexts of autoimmune diseases, graft-versus-host disease, transplant rejection, and hypersensitivity reactions.^3,4^ However, in all cases, the manufacture of clinically relevant, genetically modified CAR T cells is cumbersome and challenging. Cell preparation often takes several weeks, which restricts the use of this approach in patients with rapidly progressing disease.^5^ Furthermore, it is difficult to generate sufficient doses of clinically relevant CAR-T cells from patients who have been extensively treated with chemotherapeutic and antineoplastic drugs. The use of allogenic T cells is currently being explored but requires additional treatments to avoid graft-versus-host disease.^6^ In addition to the challenges of producing CAR T cells quickly, the use of these T cells is also associated with adverse events, such as cytokine release syndrome and neurotoxicity, and their clinical efficacy is limited by exhaustion.^7^ A novel alternative approach to CAR T-cell immunotherapy would involve the in vitro generation of genetically modified T-cell progenitors that could differentiate into naïve T cells upon transplantation into the patient. In a completely different clinical setting, poor or delayed *naïve* T-cell reconstitution following allogeneic hematopoietic stem cell transplantation (HSCT) is a limitation that exposes the patient to an elevated risk of severe and frequently lethal infections.^8–11^ In this context, the cotransplantation of human T-lymphoid progenitors (HTLPs) and hematopoietic stem and progenitor cells (HSPCs) from a conventional graft might overcome this issue by accelerating T-cell reconstitution. In light of the above information, there is an urgent need to develop a cell culture platform that can produce large numbers of clinically potent nonmodified or genetically modified immunotherapeutic HTLPs capable of rapidly reconstituting a broad subset of naïve T cells with multiple repertoires after transplantation.

In mice and humans, the early stages of T-cell development are dependent on Notch signaling (particularly signaling via delta-like ligand 4 (DL-4))^12–14^ and key cytokines involved in thymocyte survival and proliferation.^15–17^ In humans, T-cell development is typically initiated by the acquisition of surface CD7,^18^ which is followed by CD5 expression. Next, CD7- and CD5-expressing precursors give rise to a CD1a-expressing subset, which constitutes the fully committed T-cell precursors.^19^ We previously implemented a stromal cell-free culture system based on immobilized DL-4 (Notch ligand) to generate CD7^+^ HTLPs from either cord blood (CB) HSPCs or mobilized peripheral blood (mPB) HSPCs during a 7-day period of culture.^20,21^ These progenitors displayed the phenotype and molecular signature of very immature thymic progenitors and expressed T-lineage transcriptional regulators (TCF7, IL7Rα, BCL11B, GATA3, and CD3ε). Upon transplantation into irradiated adult or nonirradiated neonate NOD/SCID/γC^−/−^ (NSG) recipients, HTLPs seeded the thymus and generated mature, polyclonal and functional T cells.^20,21^ Cotransplantation of HTLPs generated in vitro and nonmanipulated CD34^+^ HSPCs led to robust, rapid reconstitution of the T-cell compartment, while the other hematopoietic lineages arose from the nonmanipulated HSPCs.^20^

Mobilized peripheral blood is currently the main source of HSPCs for allogenic HSCT due to its availability in large quantities. Although mPB HSPCs have several advantages over CB HSPCs in clinical settings, they are less able to differentiate into lymphoid lineages (particularly the T-cell lineage).^22^ Moreover, fewer HTLPs were generated by culturing mPB HSPCs in our immobilized DL-4 system than by culturing CB HSPCs, possibly due to intrinsic differences in T-cell potential, survival and proliferation. This low yield was accompanied by a higher apoptosis rate, a lower proliferation rate, and less efficient, delayed reconstitution of mature T cells upon transplantation into NSG mice.^21^ Therefore, we decided to screen for potent molecules that could improve the cell expansion and yield of HTLPs in our culture system, with a main focus on mPB. Based on our literature search, we selected StemReginin 1 (SR1) (an aryl hydrocarbon receptor antagonist), UM171 and UM729 (pyrimidoindole derivatives), tumor necrosis factor alpha (TNFα) and 2-phospho-L-ascorbic acid (Ph-AA) for screening. SR1, UM171 and UM729 are known to promote human hematopoietic stem cell (HSC) expansion.^23–25^ In contrast, Ph-AA and TNFα were previously reported to promote the in vitro T-cell potential of human HSCs and HSPCs.^26–28^ We found that the addition of TNFα dramatically increased the yield of CD7^+^ HTLPs derived from either CB HSPCs or adult mPB HSPCs and the purity of the final cell product (an average of >80% of CD7^+^ cells for CB and >70% for mPB). We further demonstrated that this increase in yield was due to greater cell proliferation and enhanced cell survival (through decreased apoptosis) of CD7^+^ HTLPs specifically. Surprisingly, TNFα also enhanced the in vitro and in vivo T-cell potential of CB or mPB HTLPs.

Even though TNFα has an antiviral effect, we further demonstrated that (i) CB or mPB HSPCs could be transduced in a TNFα-supplemented DL-4 culture system to generate genetically modified CB or mPB HSPC-derived T-cell progenitors and (ii) the addition of TNFα enhanced the yields of transduced HTLPs by factors of 4.3- and 10.5-fold for CB and mPB, respectively. Last, we demonstrated the enhanced in vitro and in vivo T-cell potential of TNFα-exposed transduced HTLPs.

Thus, our TNFα-supplemented DL-4 culture system constitutes an efficient platform for the in vitro production of functional nonmodified or genetically modified HTLPs that possess the clinical potential to accelerate T-cell reconstitution after HSCT (to be tested in the forthcoming clinical trial NCT03879876) and to be effective as a CAR T-cell-based immunotherapy for treating a variety of diseases.

## Materials and methods

### Human samples

Following the provision of informed consent, umbilical CB samples were collected via ethically approved procedures from donors at Saint Louis Hospital (Paris, France). The mPB samples used were the unused fraction of grafts from healthy donors mobilized with granulocyte colony-stimulating factor who provided informed consent for research use. The mPB samples were part of a collection authorized by the French Ministry of Research (reference: DC-2014-2272, dated March 23rd, 2015). Cord blood and mPB CD34^+^ HSPCs were magnetically enriched (purity > 93%) from CB and mPB samples, respectively, as described previously.^29^

### Cell cultures

For screening of various molecules, human CD34^+^ HSPCs isolated from CB or mPB were cultured for 7 days at a cell concentration of 2 × 10^4^/ml in DL-4-Fc fusion protein (5 µg/ml)- and RetroNectin® (25 µg/ml) (Ozyme, Saint-Quentin-en-Yvelines, France)-coated wells of 24-well plates containing α-MEM (Gibco, Life Technologies, Carlsbad, CA) supplemented with 20% defined fetal bovine serum (HyClone, Thermo Fisher Scientific, Illkirch, France) and human cytokines (100 ng/ml interleukin (IL)-7, 100 ng/ml FMS-like tyrosine kinase 3 ligand (Flt3-L), 100 ng/ml stem cell factor (SCF) and 100 ng/ml thrombopoietin (TPO); all from PeproTech Inc., Rocky Hill, NJ)^20,21^ in the presence or absence of 100 µM Ph-AA (Sigma‐Aldrich, St. Louis, MO, USA), 750 nM SR1 (Cayman Chemical, Ann Arbor, Michigan, United States), 35 nM UM171, 1 µM U729 (STEMCELL Technologies, Vancouver, Canada) or 100 ng/ml TNFα (R&D Systems, Minneapolis, MN).

For cell cultures with or without TNFα used to generate HTLPs, human CD34^+^ HSPCs isolated from CB or mPB were cultured for 7 days at a cell concentration of 2 × 10^4^/ml in DL-4-Fc fusion protein (5 µg/ml)- and RetroNectin® (25 µg/ml) (Ozyme, Saint-Quentin-en-Yvelines, France)-coated wells of 24- or 6-well plates containing α-MEM (Gibco, Life Technologies, Carlsbad, CA) supplemented with 20% defined fetal bovine serum (HyClone, Thermo Fisher Scientific, Illkirch, France) and human cytokines (100 ng/ml interleukin 7 (IL)-7, 100 ng/ml FMS-like tyrosine kinase 3 ligand (Flt3-L), 100 ng/ml stem cell factor (SCF) and 100 ng/ml thrombopoietin (TPO); all from PeproTech Inc., Rocky Hill, NJ)^20,21^ in the presence or absence of 100 ng/ml TNFα (R&D Systems, Minneapolis, MN).

For cell cultures with/without DL-4, CD34^+^ CB or mPB HSPCs were cultured for 7 days at a cell concentration of 2 × 10^4^/ml in wells of 24-well plates coated with either the DL-4-Fc fusion protein (5 µg/ml) and RetroNectin® (25 µg/ml) or the Fc protein and RetroNectin® (25 µg/ml) in α-MEM (Gibco, Life Technologies, Carlsbad, CA) supplemented with 20% defined fetal bovine serum (HyClone, Thermo Fisher Scientific, Illkirch, France) and human cytokines (100 ng/ml IL-7, 100 ng/ml Flt3-L, 100 ng/ml SCF and 100 ng/ml TPO; all from PeproTech Inc., Rocky Hill, NJ) in the presence of 100 ng/ml TNFα (R&D Systems, Minneapolis, MN).

### DL-4 cultures of HSPC subsets

CD34^+^ CD38^+^ cells, hematopoietic stem cells, multilymphoid progenitors (MLPs), and multipotent progenitors (MPPs) were sorted from CB CD34^+^ HSPCs. The sorted HSPC subsets were cultured for 7 days in DL-4-Fc fusion protein- (5 µg/ml) and RetroNectin® (25 µg/ml) (Ozyme)-coated wells of 96-well plates (4000 cells/well) containing α-MEM (Gibco) supplemented with 20% defined fetal bovine serum (HyClone) and human cytokines (100 ng/ml IL-7, 100 ng/ml Flt3-L, 100 ng/ml SCF and 100 ng/ml TPO; all from PeproTech Inc.) in the presence or absence of 100 ng/ml TNFα (R&D Systems) (i.e., DL-4 culture conditions with or without TNFα).

### DL-4 cultures with an NFκB inhibitor

mPB CD34^+^ HSPCs were cultured in DL-4 conditions without TNFα for 5 h. An NFκB inhibitor, piceatannol (R&D Systems), was added to cultures at 25 µM. After 16 h, TNFα was added at the usual concentration of 100 ng/ml. On day 3, piceatannol was removed by replacement of the culture medium with fresh medium, and TNFα-supplemented DL-4 culture was continued to day 7.

### Transduction

CB or mPB CD34^+^ HSPCs were preactivated overnight on DL-4- and RetroNectin®-coated wells containing X-vivo 20 medium in the presence of DL-4 culture cytokines and IL-3. The cells were then transduced under the same conditions with VSV-G pseudotyped lentiviruses encoding a GFP reporter protein at a multiplicity of infection (MOI) of 100. The transduced cells were then washed with α-MEM (Gibco) and cultured for a total of 7 days in the DL-4 culture system in the presence or absence of TNFα.

### Flow cytometric analysis

Anti-human CD4-APC (clone REA623), CD4-APCVio770 (clone REA623), CD8-PEVio770 (clone REA734), CD8-APC (clone REA 734), CD34-APC (clone AC136), CD34-PECy7 (clone AC136), CD45-APCVio770 (clone REA747), CD45-PEVio770 (clone 747), CD133/1-Viobright FITC (AC133), CD3e-PE (intracellular) (clone REA975), GATA3-APC (clone REA174), CD120a (TNFRI)-PEVio770 (clone REA252), CD120b (TNFRII)-PE (clone REA520) and lineage cocktail-PE antibodies and 7-aminoactinomycin D (7-AAD) were obtained from Miltenyi Biotech (Bergisch Gladbach, Germany). Anti-human CD7-FITC (clone MT701), CD7-PE (clone MT701), CD5-PE (clone UCHT2), CD38-APC (HIT2), CD90-PECy5 (5E10), NFkB p65 (pS529)-PE (clone K10-895.12.50) and Ki-67-PECy7 (clone B56) were purchased from BD Biosciences (San Jose, CA). Anti-human CD45-BV510 (clone H130), anti-CD3-BV421 (UCHT1), anti-CD45RA-BV421 (HI100) and anti-murine CD45-BV510 (clone 30F-11) antibodies were purchased from Sony Biotechnology (San Jose, CA). Anti-human CD34-APCCy7 (581), TCRαβ-APC (IP26A), TCRαβ-PECy5 (IP26A) and Bcl-2-PE (clone 100) antibodies were purchased from BioLegend (San Diego, CA). Anti-human TCRγδ-FITC (IMMU510) and TCRγδ-PE (IMMU510) were purchased from Beckman Coulter (Brea, CA). An anti-human Bcl-11b (Ctip2)-FITC antibody (clone 25B6) was purchased from Abcam (Cambridge, UK), and an anti-human Mcl-1-Alexa Fluor 647 antibody (clone LVUBKM) was purchased from eBioscience (San Diego, CA).

For surface staining, cells were incubated with the appropriate antibodies for 15 min on ice, washed and then resuspended in FACS buffer.

For intracellular staining, cells were prestained for surface markers, fixed and permeabilized using either the Fixation/Permeabilization Solution Kit (BD Biosciences) or the Foxp3/Transcription Factor Staining Buffer Set (eBioscience) according to the manufacturer’s instructions and then incubated with the appropriate antibodies for 30 min at room temperature. The cells were then washed and resuspended in FACS buffer before analysis.

All flow cytometry data were acquired with a MACSQuant® analyzer (Miltenyi Biotech), a Gallios flow cytometer (Beckman Coulter, Krefeld, Germany) or a BD LSRFortessa™ X-20 cell analyzer (BD Biosciences) and then analyzed using FlowJo software (version 10.2, TreeStar, Ashland, OR). During FACS analyses, all gatings were performed on live cells (determined by exclusion of the dye 7-AAD).

CD34^+^CD38^+^ cells, hematopoietic stem cells, MLPs and MPPs were sorted from CB CD34^+^ HSPCs, and CD34^+^CD7^+^ and CD34^−^CD7^+^ cells were sorted after 7 days of culture on DL-4 in the presence or absence of TNFα (to exclude CD34^−^CD7^−^ and CD34^+^CD7^−^ cells from subsequent analyses). A FACSAria II SORP cell sorter (BD Biosciences) in the four-way high purity mode was used for this sorting.

### Apoptosis assay

Cells harvested from 7-day DL-4 HSPC cultures in the presence or absence of TNFα were incubated with antibodies against CD34 and CD7 on ice for 15 min. The cells were then washed and resuspended in binding buffer. Next, the cells were incubated with annexin V-PE (BD Biosciences) and 7AAD (BD Biosciences) in the dark at room temperature for 15 min and analyzed (using FACS) within an hour.

### Cell proliferation assay

CD34^+^ HSPCs from CB or mPB were labeled with CFSE using a CellTrace^TM^ CFSE kit (Life Technologies) according to the manufacturer’s instructions. The labeled cells were then cultured in the DL-4 culture system in the presence or absence of TNFα. The CFSE labeling intensity was measured daily from day 3 to day 7 after surface staining with antibodies against CD34 and CD7.

### Cell cycle assay

Cells harvested from 7-day DL-4 HSPC cultures in the presence or absence of TNFα were fixed and permeabilized (using the PerFix-nc Kit (Beckman Coulter, Brea, CA)) at room temperature for 15 min after surface staining with antibodies against human CD34 and CD7. The cells were then stained with Hoechst 33342 (Life Technologies) and an anti-Ki67-PECy7 antibody (BD Biosciences) at room temperature for 15 min.

### NFκB activation assay

mPB CD34^+^ HSPCs were seeded in DL-4- and RetroNectin-coated wells of a 96-well plate. After overnight culture, 100 ng/ml TNFα was added, and the cells were fixed with Fix Buffer 1 (BD Biosciences) after 15 min, 30 min, 60 min, 24 h or 48 h of TNFα treatment according to the manufacturer’s instructions. After fixation, the cells were then permeabilized with Perm Buffer III (BD Biosciences) according to the manufacturer’s instructions. Then, they were stained with an anti-human NFκB p65 (pS529) antibody for 3 h at room temperature. The stained cells were then washed and resuspended in FACS buffer for data acquisition on a Gallios flow cytometer (Beckman Coulter).

### RNA sequencing

RNA-seq was performed on FACS-sorted CD34^+^CD7^+^ and CD34^−^CD7^+^ progenitors on day 7 of DL-4 culture. After measurement of the RNA integrity number and removal of ribosomal RNA (using InDA-C technology, NuGEN, CA), RNA-seq libraries were generated with an Ovation RNA-seq kit (NuGEN). Library fragment size was measured with a fragment analyzer (AATI, Agilent, CA). Libraries were sequenced using a HiSeq 2500 system (Illumina, CA).

### Bioinformatic analysis of RNA-seq data

RNA reads were demultiplexed into FASTQ files, mapped to the ENSEMBL Human (hg19) reference database (using HISAT2), and then counted using featureCounts from the subread package in R. Read counts were normalized, and the subsets of progenitors induced in the presence or absence of TNFα were compared using three independent, complementary methods: DEseq2, EdgeR, and LimmaVoom. The results produced by each method were compared, grouped, and plotted as Venn diagrams. The results were filtered with the thresholds *p* ≤ 0.05 and fold change >1.2. Functional data were obtained by IPA (Qiagen) and GSEA. Heat maps were generated using the Cluster and Tree Conversion (ctc) R package and imaged using Java TreeView software (version 1.1; https://sourceforge.net/). Principal component analysis was performed with the rgl R package.

### T-cell receptor rearrangement analysis

T-cell receptor δ, γ and β rearrangement analysis was performed by multiplex PCR followed by GeneScan analysis as described previously.^30^

### Vector copy number analysis

The vector copy number was determined using a Droplet Digital PCR (ddPCR) system (Bio-Rad, Hercules, CA, USA) with specific primers and probes for HIV and albumin sequences.

### In vitro T-cell differentiation assay

The in vitro T-lymphoid potential of HTLPs or transduced HTLPs was assessed by using an OP9-hDL-1 coculture system, as described previously.^16^ CD7^+^ HTLPs or transduced HTLPs generated in 7-day DL-4 cultures of CB or mPB HSPCs in the presence or absence TNFα were cocultured on OP9-hDL1 stromal cells for 4 weeks. Every week, a quarter of the cultured cells was analyzed (using FACS) for the presence of CD4^+^CD8^+^ and TCRαβ- or TCRγδ-expressing CD3^+^ T cells; three-quarters of the cells were reseeded on fresh OP9-hDL1 cells.

### Adoptive transfer of in vitro-generated HTLPs into newborn NSG mice

A total of 5 × 10^5^ CD7^+^ HTLPs or transduced HTLPs generated in 7-day DL-4 cultures of CB or mPB HSPCs in the presence or absence of TNFα were intrahepatically transplanted into newborn NSG mice (age: 1 and 4 days). Four weeks after transplantation, the thymus was analyzed (using flow cytometry) for human cell engraftment and thymopoiesis. At this time point, human cells were undetectable in the other hematopoietic organs. The study protocol was approved by the French Ministry of Higher Education and Research (reference: APAFIS 2010-2015090411495178v4, dated November 2nd, 2015).

### Statistical analysis

The results of apoptosis experiments were analyzed using the Mann–Whitney rank-sum test. All other data were analyzed with one-way or one-way repeated-measures (RM) analysis of variance (ANOVA) or a paired or an unpaired two-tailed t test. The data were plotted as the mean ± SEM using GraphPad Prism software. The threshold for statistical significance was set to *p* < 0.05. All statistical analyses were performed using either SigmaStat software (version 3.5, Jandel Scientific, San Rafael, CA) or GraphPad Prism software (version 8.4.3, San Diego, CA).

## Results

### TNFα increases the production of DL-4-induced CD34^−^CD7^+^ progenitors

**Fig. 1:**
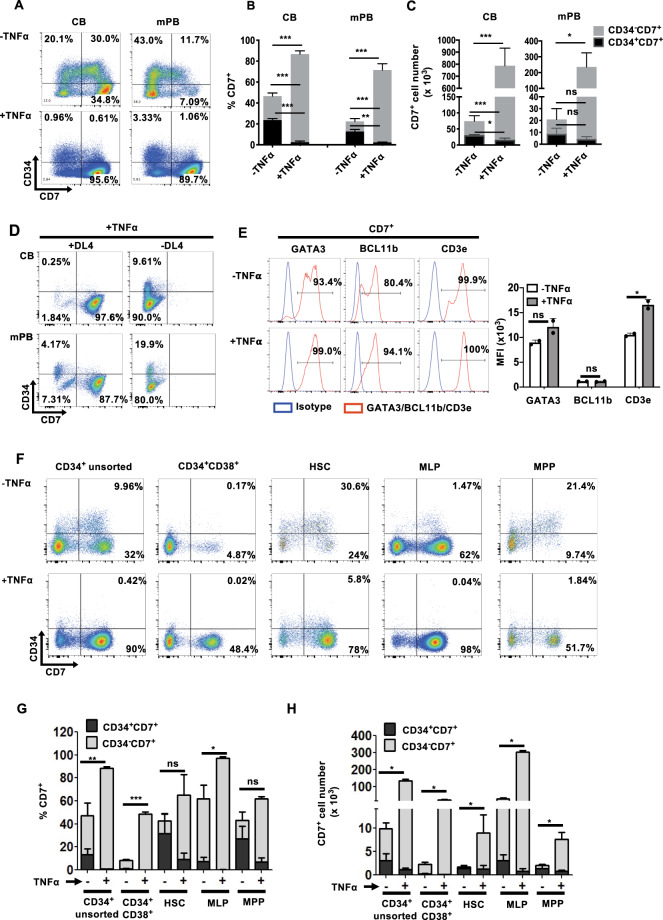
TNFα promotes the in vitro production of CD7^+^ HTLPs from DL-4 cultures of CB or mPB HSPCs. **A** A representative FACS plot of the phenotype of DL-4-cultured CB or mPB HSPCs after 7 days of culture in the presence (100 ng/ml) or absence of TNFα. Graphs showing the mean frequencies (**B**) and numbers (**C**) of CD34^+^CD7^+^ progenitors (in black) and CD34^−^CD7^+^ progenitors (in light gray) after 7 days of culture in the presence (100 ng/ml) or absence of TNFα (mean ± SEM, *n* = 7). The *p* values were calculated by one-way RM ANOVA: *p ≤ 0.05; **p ≤ 0.01; ***p ≤ 0.001. **D** Representative FACS plot of the phenotype of CB or mPB HSPC cultures with or without DL-4 in the presence of TNFα. **E** Representative FACS histograms for the expression of the T-cell commitment markers GATA3, BCL11b, and CD3ε on day-7 CD7^+^ HTLPs (left panel) and their corresponding median fluorescence intensity (MFI) values (right panel) (mean ± SEM, *n* = 2). The *p* values were calculated using an unpaired two-tailed *t* test: *p ≤ 0.05. **F** Representative flow cytometry plots of CB-derived HSPC subpopulations grown for 7 days in a DL-4 culture system in the presence or absence of TNFα. **G** Graphs showing the frequencies and **H** absolute numbers of CD34^+^CD7^+^ and CD34^−^CD7^+^ cells (mean ± SEM, *n* = 3). The *p* values were calculated by one-way ANOVA: *p ≤ 0.05; **p ≤ 0.01; ***p ≤ 0.001

First, we screened PhAA, SR1, TNFα, UM171 and UM729 for their ability to improve the cell expansion and yields of HTLPs by culturing CB or mPB CD34^+^ HSPCs with each of these molecules in a DL-4/RetroNectin® culture system (referred to henceforth as “HTLP culture”)^20,21^ for 7 days. We observed that the addition of TNFα gave rise to the highest frequency (>90%) and number (17-fold and 25-fold increases for the CB and mPB, respectively, with respect to those obtained from negative control culture without any small molecules) of CD7^+^ HTLPs among all the molecules tested (Supplementary Fig. S1A and B). Therefore, we selected TNFα to further explore its potential to improve the HTLP yield of DL-4 cultures of CB or mPB HSPCs. In the absence of TNFα, and as expected on the basis of our previous results, the day-7 cell product contained a mixture of CD34^−^CD7^−^ progenitors (i.e., myeloid progenitors^20^), CD34^+^CD7^−^ cells, and CD34^+^CD7^+^ early and CD34^−^CD7^+^ late T-cell progenitors (Fig. 1A (upper panels) and B). In the presence of TNFα, the day-7 cell product was much more homogeneous (1.85-fold increase for the CB and 3.2-fold increase for the mPB), consisting mostly of CD34^−^CD7^+^ late T-cell progenitors (mean ± standard error of the mean (SEM) frequencies of 86.74 ± 2.40% and 71.42 ± 5.84% for the CB and mPB, respectively, in the presence of TNFα vs. 46.58 ± 3.06% and 22.36 ± 3.88% in the absence of TNFα) (Fig. 1A (lower panels) and B). Hence, the addition of TNFα was associated with an average 10-fold increase in the CD7^+^ HTLP yield for both CB HSPC cultures and mPB HSPC cultures (Fig. 1C). Interestingly, neither early CD34^+^CD7^+^ progenitors nor late CD34^−^CD7^+^ progenitors were observed in the 7-day TNFα-supplemented cultures without DL-4, suggesting that the effect of TNFα was dependent on DL-4 (Fig. 1D).

Daily CD34/CD7 phenotyping showed differentiation of CB and mPB HSPCs into early CD34^+^CD7^+^ progenitors but not differentiation into late CD34^−^CD7^+^ T-cell progenitors until day 4. In the presence of TNFα, however, higher levels of differentiation into late CD34^−^CD7^+^ T-cell progenitors were observed from day 5 onwards, with a frequency of nearly 90% at day 7 (Supplementary Fig. S2A).

Regardless of TNFα exposure, HTLPs barely expressed CD5 or CD1a (Supplementary Fig. S2B) or harbored any detectable T-cell receptor (TCR)δ, γ and β rearrangements (Supplementary Fig. S3A and B). Importantly, CD7^+^ HTLPs expressed GATA3, BCL11B (two key transcriptional regulators of T-cell differentiation^31^), and CD3ε (Fig. 1E). Notably, the expression level of CD3ε was significantly higher in TNFα-exposed HTLPs than in unexposed HTLPs. However, the expression levels of BCL11b and GATA3 showed no significant differences between TNFα-exposed and nonexposed HTLPs (Fig. 1E).

Next, to determine which HSPC subset responded to TNFα treatment, we used fluorescence-activated cell sorting (FACS) to isolate CD34^+^CD38^+^ cells, HSCs, multilymphoid progenitors (MLPs) and multipotent progenitors (MPPs) from CB CD34^+^ cells (Supplementary Fig. S3C) and cultured the cells in the presence or absence of TNFα. The results demonstrated that both the frequencies and absolute counts of day-7 CD7^+^ progenitor cells (i.e., HTLPs) obtained from these subpopulations were higher in the presence of TNFα than in its absence (Fig. 1F–H). Interestingly, MLPs and CD34^+^CD38^+^ cells appeared to have the greatest potential for generating late T-cell progenitors (CD34^−^ CD7^+^) in the presence of TNFα during the culture period of 7 days.

Taken together, these data show that TNFα accelerates and increases the production of CD34^−^CD7^+^ progenitors from all HSPC cell subsets without any restriction to a specific progenitor subset.

### TNFα specifically promotes the survival and proliferation of CD7^+^ progenitors

**Fig. 2:**
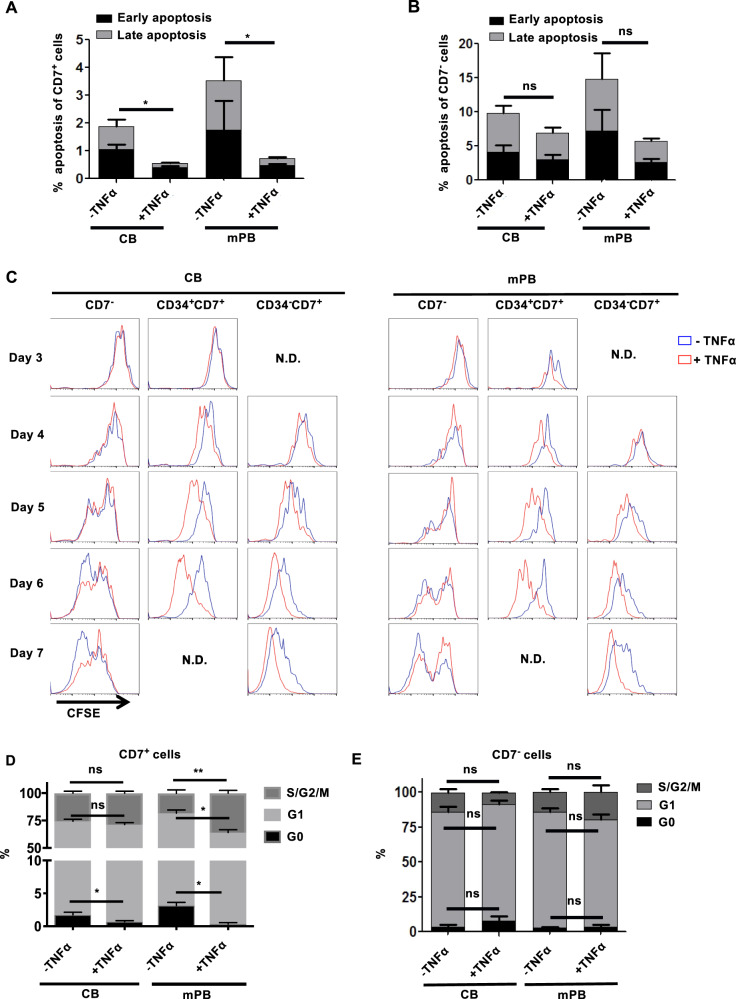
Enhanced cell survival and proliferation of CD7^+^ HTLPs in the presence of TNFα in DL-4 cultures of CB or mPB HSPCs. **A**,**B** The level of apoptosis, analyzed by staining with Annexin-V and 7-AAD (early and late apoptosis correspond to Annexin-V^+^7-AAD^−^ and Annexin-V^+^7-AAD^+^ phenotypes, respectively), among CD7^+^ HTLPs (**A**) and CD7^−^ cells (**B**) after 7 days of DL-4 culture in the presence or absence of TNFα (mean ± SEM, *n* = 4). *P* values were calculated using the Mann–Whitney rank-sum test; *p ≤ 0.05. **C** Assessment of cell proliferation (based on CFSE dilution) from day 3 to day 7 in DL-4 cultures in the presence or absence of TNFα. CD34^+^ cells were labeled with CFSE prior to DL-4 culture. The frequencies of quiescent (G0) cells and cells in the G1 or S/G2/M phases of the cell cycle among CD7^+^ HTLPs (**D**) and CD7^−^ cells (**E**) after 7 days of DL-4 culture in the presence or absence of TNFα (mean ± SEM, *n* = 3). The *p* values were calculated by one-way RM ANOVA: *p ≤ 0.05; **p ≤ 0.01

To explore the mechanism underlying the TNFα-associated increase in the generation of CD34^−^CD7^+^ progenitors, we assessed the apoptosis and proliferation of HTLPs in HTLP cultures. Staining with Annexin V and 7-aminoactinomycin D (7AAD) showed that compared with no exposure, TNFα exposure was associated with a significantly lower level of apoptosis among CD7^+^ progenitors in HTLP cultures of CB and mPB HSPCs (Fig. 2A). In contrast, the level of apoptosis among CD7^−^ cells was slightly but not significantly lower in both CB HSPC cultures and mPB HSPC cultures in the presence of TNFα (Fig. 2B). Moreover, flow cytometric analyses for the expression of Bcl-2 and Mcl-1 (antiapoptotic proteins^32,33^) revealed that although all CD7^+^ progenitors expressed Bcl-2 in both culture conditions (with or without TNFα), the addition of TNFα was associated with an increase in the proportion of Mcl-1-expressing cells among the CD7^+^ progenitors (Supplementary Fig. S4A and B). Importantly, the levels of expression of both Bcl-2 and Mcl-1 in CD7^+^ progenitors were significantly higher in cultures with TNFα (Supplementary Fig. S4A and B). These results suggested that TNFα might increase the survival of CD7^+^ progenitors by upregulating Bcl-2 and Mcl-1 expression and thus protecting the cells from apoptosis.

The results of a cell proliferation assay (based on the dilution of carboxyfluorescein succinimidyl ester (CFSE) labeling) showed that although the CD7^−^ cell proliferation rate was least affected by TNFα exposure, the proliferation rates of both CD34^+^CD7^+^ T-cell progenitors and CD34^−^CD7^+^ T-cell progenitors were increased in the presence of TNFα (starting from day 4 and day 5 of HTLP culture, respectively; Fig. 2C). These observations were in line with the results of a cell cycle assay (with Hoechst 33342 and anti-Ki67 antibody staining) performed with HTLP cultures; a higher proportion of S/G2/M-phase cells and a lower proportion of G0-phase cells were observed among CD7^+^ cells (especially for mPB cultures) in the presence of TNFα, while the proportions were similar for CD7^−^ cells (Fig. 2D,E). Taken together, these data demonstrate that TNFα specifically (i) increases the survival of CD7^+^ progenitors by decreasing apoptosis and (ii) enhances the proliferation of CD7^+^ progenitors by increasing cell cycling (possibly through the NFkB signaling pathway^34^).

### Transcriptomic profiles of CB- and mPB-derived HTLPs and the role of NFκB signaling in TNFα-induced enhanced HTLP generation

**Fig. 3:**
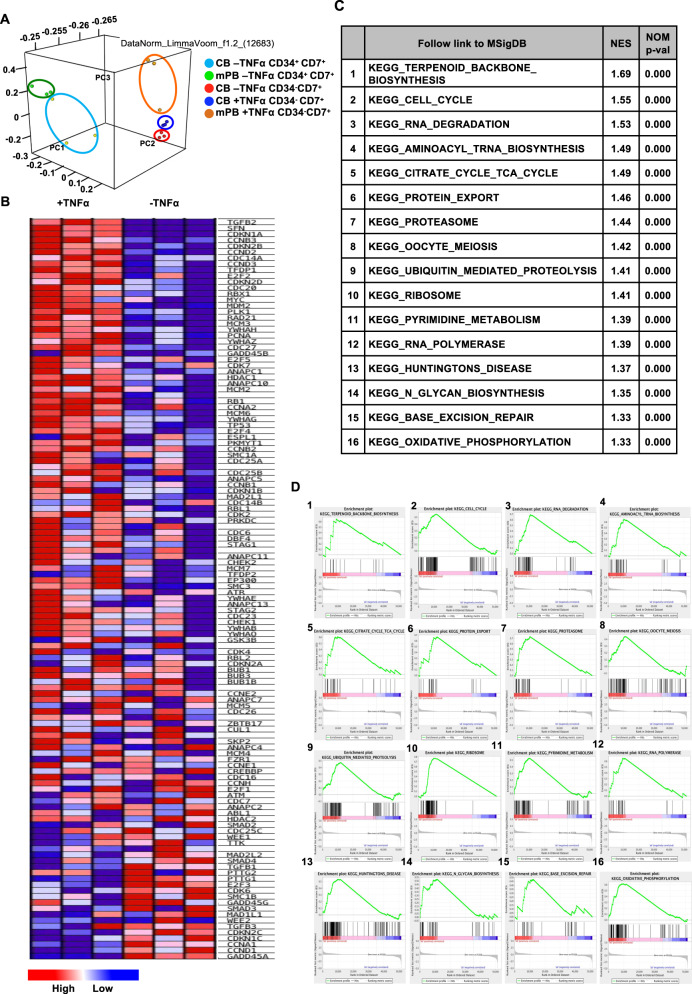
Similar transcriptomic profiles of TNFα-exposed and nonexposed CD34^−^CD7^+^ late T-cell progenitors. **A** PCA after RNA sequencing of sorted CB or mPB HSPC-derived CD34^+^CD7^+^ and CD34^−^CD7^+^ progenitors after 7 days of DL-4 culture. Each point represents a replicate sample. **B** A heat map representing the gene expression profile for the cell cycle pathway among CD34^−^CD7^+^ progenitors generated from CB HSPCs. **C**, **D** GSEA of RNA-seq data obtained from CD34^−^CD7^+^ cells generated in the presence or absence of TNFα. **C** The 16 pathways (out of 20) with the highest normalized enrichment scores (all *p* < 0.01) and **D** their corresponding GSEA plots, showing the enrichment profile

**Fig. 4:**
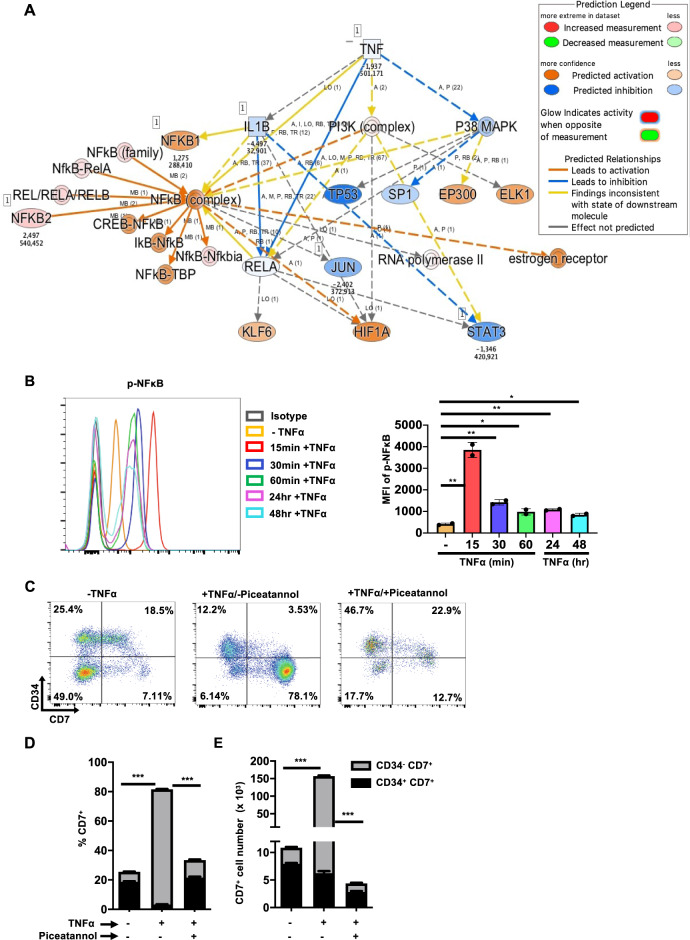
NFκB signaling mediated TNFα-induced enhanced CD7^+^ HTLP generation. **A** The predicted regulatory pathway network by IPA based on differential gene expression by CD34^−^CD7^+^ cells generated in the presence or absence of TNFα. IPA was performed after RNA sequencing of sorted CB or mPB HSPC-derived CD34^+^CD7^+^ and CD34^−^CD7^+^ progenitors after 7 days of DL-4 culture. **B** A representative FACS histogram showing the phosphorylation of NFκB (left panel) and corresponding MFIs (right panel) at the indicated time points after treatment with TNFα during HTLP culture (mean ± SEM, *n* = 2). The *p* values were calculated by an unpaired two-tailed *t* test: *p ≤ 0.05; **p ≤ 0.01. **C** A representative FACS plot of the phenotype of 7-day HTLP cultures of mPB HSPCs in the presence (100 ng/ml) or absence of TNFα or in the presence of TNFα and piceatannol (25 µM) (an NFkB inhibitor). Graphs showing the mean frequencies (**D**) and numbers (**E**) of CD34^+^CD7^+^ progenitors (in black) and CD34^−^CD7^+^ progenitors (in light gray) after 7 days of HTLP culture of mPB HSPCs in the presence (100 ng/ml) or absence of TNFα or in the presence of TNFα and piceatannol (25 µM) (an NFkB inhibitor) (mean ± SEM, *n* = 3). The *p* values were calculated by one-way ANOVA: ***p ≤ 0.001

To explore the effect of TNFα on the transcriptomic profile of day-7 HTLPs, we sequenced and compared RNA from late (CD34^−^CD7^+^) progenitors produced in TNFα-supplemented HTLP cultures and from early (CD34^+^CD7^+^) and late progenitors produced in the absence of TNFα. Unfortunately, the numbers of late progenitors obtained with mPB HSPCs in the absence of TNFα and the numbers of early progenitors obtained in the presence of TNFα were very low, which prevented us from assessing gene expression levels. Principal component analysis of gene expression showed a clear difference in the clustering of early (CD34^+^CD7^+^) vs. late (CD34^−^CD7^+^) progenitors under all conditions (Fig. 3A). Notably, the late (CD34^−^CD7^+^) progenitors derived from TNFα-supplemented or nonsupplemented CB HTLP cultures were found to be closely clustered, suggesting that TNFα treatment did not alter the progenitor transcriptomic profile (Fig. 3A). Moreover, the clustering of TNFα-exposed mPB late progenitors and TNFα-exposed and nonexposed CB late progenitors indicated similar transcriptomic profiles, regardless of the cell source (Fig. 3A). When we performed gene set enrichment analysis (GSEA) of FACS-sorted CD7^+^ T-cell progenitors, we observed a clear enrichment in cell cycle pathways in the presence of TNFα (*p* < 0.01; Fig. 3B–D). This agreed with the observed effect of TNFα on the cell cycle, as described above (Fig. 2D). Ingenuity pathway analysis (IPA) of the RNA-sequencing (RNA-seq) results predicted that the NFκB signaling pathway must be activated by TNFα to explain the difference in the gene expression profiles (Fig. 4A). This result is in line with literature reports showing that TNFα is one of the main activators of the NFκB signaling pathway.^35^

We showed that tumor necrosis factor receptors (TNFRs) I and II were expressed in HSPCs (Supplementary Fig. S5A). Interestingly, TNFα significantly and specifically increased the expression of both TNFR I and TNFR II in CD7^+^ HTLPs (Supplementary Fig. S5B and C, left panels) compared to CD7^−^ cells (Supplementary Fig. S5B and C, right panels). Next, we explored the activation of NFκB signaling by TNFα in HTLP cultures. As shown in Fig. 4B, there was a low basal level of phosphorylated NFκB in HTLPs without TNFα treatment. However, TNFα tremendously increased NFκB signaling after 15 min. Between 15 and 60 min, the level of phosphorylated NFκB decreased slowly and then remained stable up to 48 h at a level higher than the basal level.

To further confirm that the TNFα-mediated effect on CD7^+^ cells was – at least partly – a consequence of the activation of NFκB signaling, mPB HSPCs were cultured under HTLP conditions in the presence or absence of TNFα or in the presence of both TNFα and piceatannol (an NFκB inhibitor). We observed that the addition of piceatannol abolished the TNFα-induced effects on both the frequency and yield of CD7^+^ HTLPs (with decreases of 2.4- and 35-fold, respectively, Fig. 4C–E), showing the importance of NFκB signaling in the TNFα-induced effect. Interestingly, the inhibition of NFκB signaling by piceatannol significantly reduced the levels of TNFR I and II expression in CD7^+^ HTLPs (Supplementary Fig. S5B and C, left panels), suggesting a role for NFκB signaling in this process.

Taken together, these data suggested that TNFα increased the generation of CD7^+^ HTLPs in HTLP cultures by activating NFκB signaling.

### Enhanced T-cell potential of TNFα-exposed HTLPs

**Fig. 5:**
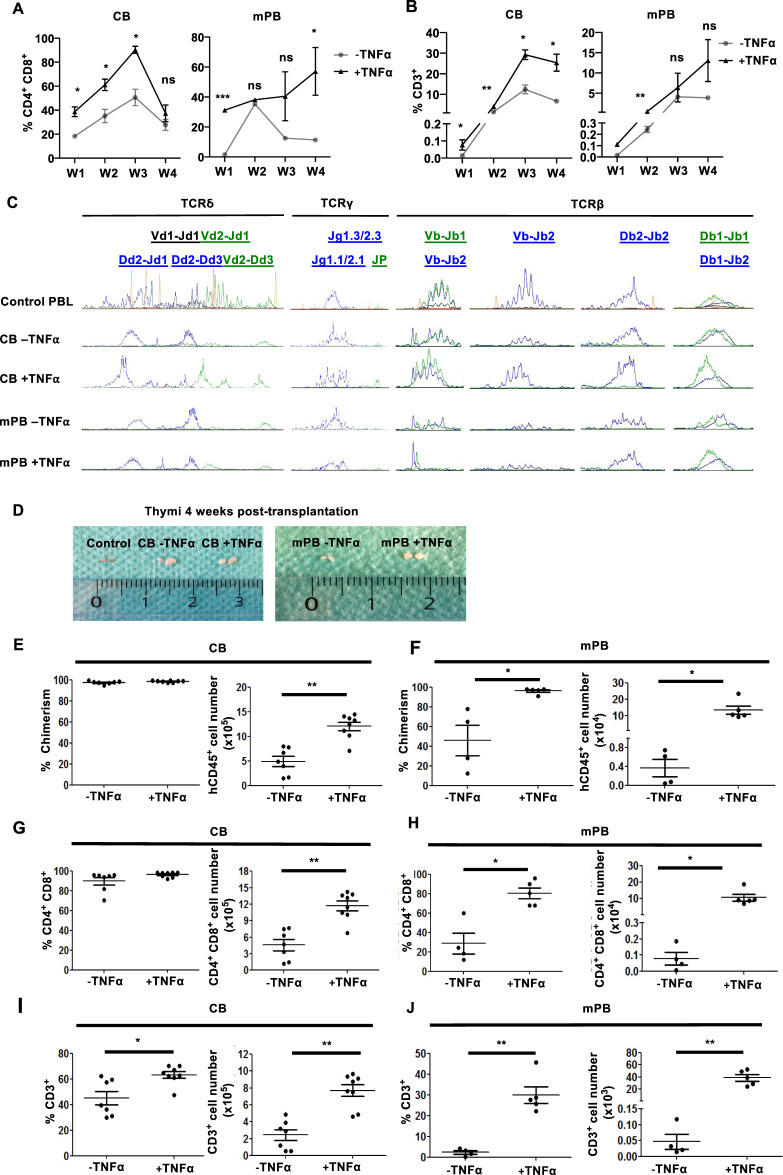
TNFα enhances the in vitro and in vivo T-cell potential of CD7^+^ HTLPs derived from CB or mPB HSPCs. Graphs showing the mean frequencies of CD4^+^CD8^+^ cells (**A**) and CD3^+^ cells (**B**) obtained after 1, 2, 3 and 4 weeks of coculture of day-7 CB or mPB HSPC-derived HTLPs (with or without TNFα treatment) with OP9-hDL1 stromal cells (mean ± SEM, *n* = 3). The *p* values were calculated using a paired two-tailed *t* test: *p ≤ 0.05; **p ≤ 0.01; ***p ≤ 0.001. **C** Analyses of TCRδ, TCRγ and TCRβ rearrangements in T cells differentiated from CB or mPB CD7^+^ HTLPs after OP9-hDL1 coculture. Each peak represents the fluorescence intensity of the corresponding rearrangement loci; positive control: peripheral blood lymphocytes (PBLs). **D** Representative photographs of the thymus four weeks after intrahepatic injection of 5 × 10^5^ CB or mPB HSPC-derived HTLPs (cultured with or without TNFα treatment) into 1- to 4-day-old NSG mice. Graphs showing the chimerism and numbers of hCD45^+^ cells in the thymus of CB (**E**) and mPB (**F**) HSPC-derived HTLP recipients. Graphs showing the frequencies and numbers of CD4^+^CD8^+^ cells for CB-HTLP (**G**) and mPB-HTLP (**H**). Graphs showing the frequencies and numbers of CD3^+^ cells for CB-HTLP (**I**) and mPB-HTLP (**J**). Each dot represents a recipient mouse. The *p* values were calculated by one^-^way RM ANOVA: *p ≤ 0.05; **p ≤ 0.01

We next tested the in vitro T-cell differentiation potential of CD7^+^ HTLPs by performing cocultures with human delta-like-1 (hDL-1)-expressing bone marrow (BM) stromal (OP9-hDL1) cells. The T-cell differentiation status was analyzed weekly during the cocultures. CD4^+^CD8^+^ double-positive (DP) cells appeared at week 1 in the CB HTLP cocultures (whether treated with TNFα or not) and in the TNFα-treated mPB HTLP coculture and at week 2 in the nontreated mPB HTLP coculture (Fig. 5A and Supplementary Fig. S6A). Moreover, CD3^+^ cells (expressing γδ or αβ TCRs) were detected earlier in TNFα-treated CB and mPB HTLP cocultures than in nontreated HTLP cocultures (Fig. 5B and Supplementary Fig. S6B). Importantly, TNFα-exposed CB and mPB HTLP cocultures gave rise to higher proportions of DP and CD3^+^ cells than nonexposed cultures (Fig. 5A, B and Supplementary Fig. S6A and B). These observations suggest that TNFα-treated CB and mPB HTLPs differentiate more rapidly and efficiently into T cells. Importantly, the TCR repertoire (i.e., δ, γ and β locus rearrangements) was polyclonal without any bias under all conditions (Fig. 5C).

The in vivo T-cell potential of TNFα-treated HTLPs was then tested by intrahepatic transplantation into nonirradiated neonatal NSG mice. Four weeks after transplantation, human (h)CD45^+^ cells colonized the thymus to a similarly high extent (>90%) in all CB HTLP (whether treated with TNFα or not) recipients. However, the hCD45^+^ cell count was 2.4-fold higher for TNFα-treated CB HTLPs than for nontreated HTLPs (Fig. 5E and Supplementary Fig. S6C). The frequencies and numbers of hCD45^+^ cells were also higher for TNFα-treated mPB HTLPs (by factors of 2.1 and 39.4, respectively) than for nontreated HTLPs (Fig. 5F and Supplementary Fig. S6C). Active thymopoiesis (measured by the presence of human CD45^+^CD4^+^CD8^+^ DP cells) was observed under all conditions (Fig. 5G,H and Supplementary Fig. S6D). In CB HTLP recipients, although the proportion of DP cells was similar, the number of DP cells was 2.5-fold higher for TNFα-treated HTLPs (Fig. 5G and Supplementary Fig. S6D). In recipients of mPB HTLPs, the frequencies and numbers of DP cells were greater for TNFα-treated HTLPs (by factors of 2.8 and 134, respectively) than for untreated HTLPs (Fig. 5H and Supplementary Fig. S6D); this finding was consistent with the observed enlargement of the thymus (Fig. 5D). The recipients of TNFα-treated HTLPs, relative to untreated HTLP recipients, showed enhanced CD3^+^ cell frequencies (1.4- and 12.3-fold increases for CB and mPB HTLPs, respectively) and numbers (3- and 768-fold increases for CB and mPB HTLPs, respectively) (Fig. 5I,J and Supplementary Fig. S6E). These results demonstrate the enhanced in vitro and in vivo T-cell potential of TNFα-treated HTLPs derived from CB or mPB HSPCs.

### Efficient production of transduced genetically modified HTLPs from TNFα-supplemented HTLP cultures

**Fig. 6:**
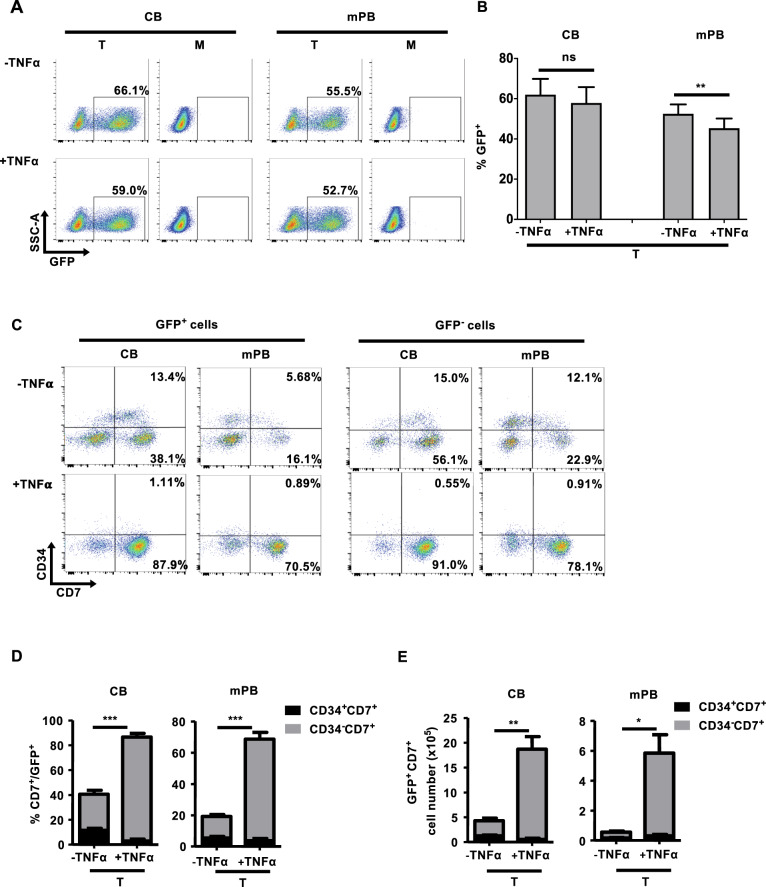
Efficient generation of transduced HTLPs from TNFα-supplemented DL-4 cultures of CB or mPB HSPCs. **A** A representative FACS plot of the transduction efficiency of CB or mPB HSPCs after transduction (T) or nontransduced mock (M) treatment with a GFP-encoding lentiviral vector and 7 days of DL-4 culture in the presence (100 ng/ml) or absence of TNFα. **B** Graph showing transduction efficiencies (mean ± SEM, *n* = 5). **C** A representative FACS plot of the phenotype of transduced (GFP^+^) (left panel) and nontransduced (GFP^−^) (right panel) cells, showing CD34^+/−^CD7^+^ T-cell progenitor differentiation. Graphs showing the mean frequencies (**D**) and numbers (**E**) of transduced CD34^+/−^CD7^+^ progenitors after 7 days of culture under transduction conditions in the presence (100 ng/ml) or absence of TNFα (mean ± SEM, *n* = 5). The *p* values were calculated by one-way RM ANOVA: *p ≤ 0.05; **p ≤ 0.01; ***p ≤ 0.001

To examine whether genetically modified transduced CD7^+^ HTLPs could be generated in TNFα-supplemented HTLP cultures, CB or mPB CD34^+^ HSPCs were preactivated, transduced or not (mock) with a vesicular stomatitis virus (VSV)-G pseudotyped lentiviral vector encoding a GFP reporter on DL-4/RetroNectin® and cultured for a total of 7 days in the presence or absence of TNFα. Analysis of the transduction efficiency (based on the expression of GFP) in the HTLP cultures showed that the HSPCs could be efficiently transduced (mean ± SEM transduction efficiencies in the presence and absence of TNFα: 57.8 ± 7.89% and 61.9 ± 7.86% for CB and 45.3 ± 4.84% and 52.5 ± 4.66% for mPB, respectively) (Fig. 6A,B). Although the addition of TNFα did not significantly affect the transduction efficiencies of CB HTLP cultures, it significantly decreased the transduction efficiencies of mPB HTLP cultures by 1.2-fold (Fig. 6A,B). The transduced cells in HTLP cultures of CB or mPB HSPCs were able to differentiate into CD7^+^ HTLPs in the presence or absence of TNFα (Fig. 6C,D). Although the transduced GFP^+^ cells showed decreased CD7^+^ HTLP frequencies compared to the nontransduced GFP^−^ cells in cultures without TNFα, the CD7^+^ HTLP frequencies in GFP^+^ and GFP^−^ cells were similar in cultures supplemented with TNFα (Fig. 6C). As observed in the cultures without genetic manipulation, the frequencies of transduced CD7^+^ HTLPs were significantly higher for TNFα-supplemented cultures than for nonsupplemented cultures (mean ± SEM frequencies in the presence and absence of TNFα: 80.682 ± 2.4% vs. 36.67 ± 4.52% for CB and 68.6 ± 2.23% vs. 19.2 ± 3.63% for mPB) (Fig. 6C,D). The TNFα-induced increase in the frequency of transduced HTLPs was accompanied by a greater yield of transduced HTLPs (by factors of 4.3 and 10.5 for CB and mPB, respectively) (Fig. 6E). These data indicate that large numbers of transduced genetically modified HTLPs can be efficiently generated in vitro in TNFα-supplemented HTLP cultures of CB or mPB HSPCs.

### Enhanced T-cell potential of transduced genetically modified TNFα-exposed HTLPs

**Fig. 7:**
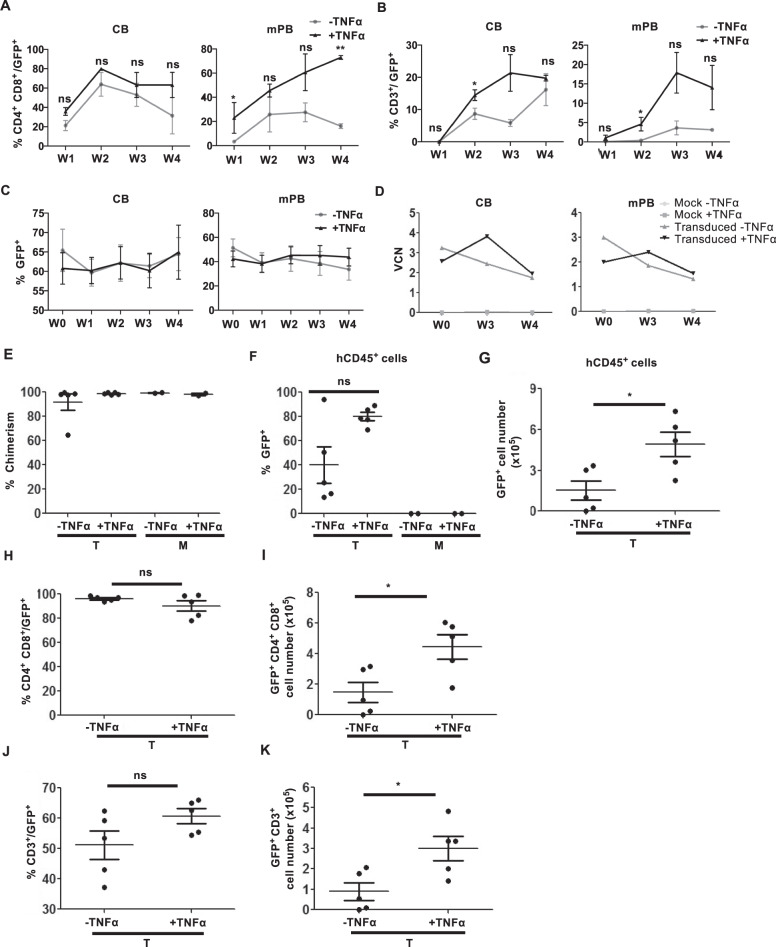
Enhanced in vitro and in vivo T-cell potential of transduced CD7^+^ HTLPs. Graphs showing the mean frequencies of transduced CD4^+^CD8^+^ cells (**A**) and CD3^+^ cells (**B**) obtained after 1, 2, 3 and 4 weeks of coculture of day-7 CB or mPB HSPC-derived transduced HTLPs (with or without TNFα treatment) with OP9-hDL1 stromal cells (mean ± SEM, *n* = 3). The *p* values were calculated using a paired two-tailed *t* test: *p ≤ 0.05; **p ≤ 0.01. **C** A graph showing the mean frequencies of transduced cells during 4 weeks of OP9-hDL1 coculture (mean ± SEM, *n* = 3). **D** A graph representing the vector copy number per cell of OP9-hDL1 cell-cocultured transduced CB or mPB HTLPs at the indicated weeks of coculture. **E** Graphs showing the chimerism of hCD45^+^ cells in the thymus of recipient NSG mice four weeks after intrahepatic injection of 5 × 10^5^ CB HSPC-derived transduced (T) or nontransduced mock (M) HTLPs (cultured with or without TNFα treatment) into 1- to 4-day-old NSG neonates. Graphs showing the frequencies (**F**) and numbers (**G**) of transduced hCD45^+^ cells in the thymus of NSG mouse recipients. Graphs showing the frequencies (**H**) and numbers (**I**) of transduced CD4^+^CD8^+^ cells in the thymus of recipient mice. Graphs showing the frequencies (**J**) and numbers (**K**) of transduced CD3^+^ cells in the thymus of recipient mice. Each dot represents a recipient mouse. The *p* values were calculated by one-way RM ANOVA: *p ≤ 0.05

Upon OP9-hDL1 coculture, transduced CD4^+^CD8^+^ DP cells appeared at week 1 in the transduced CB HTLP cocultures (regardless of TNFα treatment) and in the TNFα-treated transduced mPB HTLP coculture and at week 2 in the nontreated transduced mPB HTLP coculture (Fig. 7A and Supplementary Fig. S7A). In addition to accelerated T-cell differentiation, an elevated frequency of transduced DP cells was observed in the TNFα-treated mPB HTLP coculture compared to the nontreated counterpart (Fig. 7A and Supplementary Fig. S7A). Transduced CD3^+^ cells (expressing γδ or αβ TCRs) were detected under all conditions from week 2 onwards, with higher proportions in transduced TNFα-exposed CB or mPB HTLP cocultures than in their nonexposed counterparts (Fig. 7B and Supplementary Fig. S7B). These data suggest that transduced TNFα-treated CB and mPB HTLPs differentiate more efficiently into T cells than their untreated counterparts.

The constant expression of GFP (Fig. 7C) indicated stable transduction of HTLPs under all conditions. This observation was consistent with the stable vector copy numbers observed at week 0 (before the coculture) and at weeks 3 and 4 of coculture (Fig. 7D).

Next, we tested the in vivo T-cell potential of transduced HTLPs following intrahepatic transplantation into nonirradiated neonatal NSG mice. Four weeks after transplantation, human (h)CD45^+^ cells colonized the thymus to a similarly high extent (>90%) in all the recipients (regardless of TNFα treatment and transduction), with the exception of one of the recipients of transduced nontreated HTLPs (Fig. 7E and Supplementary Fig. S7C). The proportions of transduced hCD45^+^ cells were less varied among the TNFα-treated HTLP recipients than among nontreated HTLP recipients (Fig. 7F and Supplementary Fig. S7D). Additionally, the transduced hCD45^+^ cell count was 3.2-fold higher for TNFα-treated HTLPs than for nontreated HTLPs (Fig. 7G). Active thymopoiesis of transduced HTLPs (measured by the presence of transduced human CD45^+^CD4^+^CD8^+^ DP cells) was observed under both conditions (Fig. 7H and Supplementary Fig. S7E). Although the proportions of transduced DP and CD3^+^ cells were similar, their counts were significantly (3-fold and 3.3-fold increases, respectively) higher for TNFα-treated HTLPs (Fig. 7H–K and Supplementary Fig. S7E,F). Taken together, these data demonstrate that transduced genetically modified HTLPs can efficiently differentiate into transduced T cells both in vitro and in vivo. Furthermore, TNFα-treated HTLPs have a greater T-cell potential than nontreated HTLPs.

## Discussion

Adult HSPCs harvested from mPB are increasingly being used for related-donor and unrelated-donor HSCT, and this approach has several advantages over CB grafts.^36,37^ Adult mPB not only yields more HSPCs but can also be used to generate an autologous graft, reducing the risks of graft rejection and graft-versus-host disease. When compared with BM as a source of HSPCs, adult mPB requires does not require anesthesia, hospitalization or blood transfusion for collection; hence, the probability of a serious adverse event is very low. However, in regard to generating T-cell progenitors and thus accelerating T-cell recovery after HSCT, our previously published work highlighted a delay in T-cell progenitor commitment and a very low HTLP yield for mPB HSPCs in a DL-4 culture system.^21^ Hence, we screened a number of molecules (2-phosphate-L-ascorbic acid, StemReginin 1, UM171, UM729 and TNFα) to improve cell yields and cell expansion in the DL-4 culture system, with a focus on mPB HSPC-derived progenitors. We found that the addition of TNFα to the DL-4 culture system specifically enhanced the generation of CD7^+^ HTLPs by increasing the survival and proliferation of CD7^+^ progenitors, resulting in a highly homogeneous cell product. These HTLPs differentiated very quickly and efficiently (with even better results obtained with mPB) in vitro and in vivo.

Previously, Seet and coll. established an efficient method to generate mature T cells from multiple human HSPC sources using artificial thymic organoids under serum-free conditons.^38^ Another approach for the generation of murine and human T-cell progenitors from mouse fetal HSPCs and human CB HSPCs using immobilized DL-4 and vascular cell adhesion molecule 1 (VCAM-1) was also reported.^39^ Unlike these approaches, our TNFα-supplemented immobilized DL-4 culture system generated high numbers of HTLPs from CB or mPB HSPCs.

Tumor necrosis factor alpha has a protective effect on HSPCs. The transplantation of lineage-negative BM cells from TNF receptor-deficient mice into wild-type recipients was shown to result in defective early engraftment and the loss of a lasting hematopoietic contribution from the host.^40^ Human HSPCs obtained from BM, CB or mPB are highly resistant to TNFα-mediated apoptosis, whereas mature cells (such as CD3^+^ T cells, CD19^+^ B cells, and CD33^+^ myeloid cells) are more susceptible.^41–47^ Interestingly, some researchers have identified a link between TNFα and thymopoiesis. As the key stimulus for NFκB signaling, TNFα is expressed in the thymic microenvironment by macrophages, thymic stromal cells, and thymocytes themselves.^48,49^ It has been reported that preincubation of BM- or CB-derived CD34^+^ cells with TNFα increases the generation of mature T cells in an ex vivo murine fetal thymic culture system.^27,28^ The expression and activation of TNF receptor superfamily members, including TNFRII, as observed in our data, are also known to promote regulatory T-cell differentiation in murine thymocytes.^50^ Mechanistically, TNFα was shown to upregulate CD127 (the interleukin (IL)-7 receptor alpha-chain) in a small fraction of BM-derived CD34^+^ HSPCs and the T-cell commitment factor Gata3 in a small fraction of CB-derived CD34^+^ HSPCs. However, none of these studies examined the specific effects of TNFα during the early stages of T-cell commitment. In the present study, we demonstrated that TNFα specifically influences the proliferation and survival (and thus the frequency and yield) of early and late CD7^+^ progenitors. The effects of TNFα are DL-4 dependent and seem to rely on activation of the NFkB pathway, in line with other studies showing the positive effect of Notch on NFkB activation.^51^ These effects thus combine (i) accelerated T-cell differentiation through upregulated GATA3 and BCL11B expression, (ii) decreased apoptosis achieved by upregulating the expression of antiapoptotic molecules such as Bcl-2 and Mcl-1 in CD7^+^ cells (our data) and potentially cellular Inhibitor of Apoptosis Protein 1 or 2,^52^ and (iii) accelerated entry into the cell cycle, possibly through p53 pathway inhibition. Further investigations of the mechanisms of action involving TNFα are warranted to test these hypotheses.

We scaled up our TNFα-supplemented high-performance CD7^+^ HTLP culture system for potential use in clinical applications. Indeed, we have shown that the outcome of CD7^+^ HTLP generation in a preclinical setting (i.e., with clinical-grade reagents) is similar to that obtained in the research-grade system described in detail above. Moreover, karyotyping and in vivo tumorigenicity studies of the cell product suggest that CD7^+^ HTLPs can be used safely in the clinic. The ability of transplanted CD7^+^ progenitors generated in the present culture system to treat T-cell deficiencies will be investigated in a forthcoming clinical trial (NCT03879876) that has been approved by the French Drug Agency. Twelve SCID patients will receive haploidentical CD34^+^ HSPCs and a single dose of HTLPs. The study’s primary objective is to assess the overall safety and dose-related toxicity of this protocol. The secondary objectives include assessments of graft rejection, the presence of naïve CD4^+^ T cells at 6 months, the time course of T-cell reconstitution, the incidence of infections, the relapse rate, and overall survival.

We are also initiating a clinical trial of CB-based HSCT in adult patients with leukemia who will receive two CB grafts: one prepared in the TNFα-supplemented DL-4 culture system and one that has not been manipulated. These two trials will provide valuable information on the feasibility and safety of HTLP engraftment and the comparative advantages or disadvantages of mPB- vs. CB-derived HTLPs.

Tumor necrosis factor alpha mediates the inflammatory response;^53^ hence, agents that block TNFα activity are widely used to treat inflammatory diseases and certain autoimmune diseases (such as rheumatoid arthritis). On the other hand, tumor necrosis factor alpha is secreted in response to viral infections and has a protective role in host defense against viral infections. Not surprisingly, anti-TNFα therapy increases patient susceptibility to infection by various viruses (e.g., HIV and Epstein-Barr virus) by countering the protective antiviral effect of this cytokine.^54,55^ This factor has long been known to have antiviral activity mediated by killing infected cells directly or by increasing uninfected cell resistance to infection.^56–59^ These observations suggest that the presence of TNFα is not compatible with lentiviral transduction. In the present study, however, we were able to successfully transduce CB and mPB HSPCs under TNFα-supplemented DL-4 culture conditions and thus generate large numbers of transduced HTLPs. Furthermore, compared to nontransduced cells, transduced HTLPs were as capable of differentiating into T cells in vitro and in vivo. The combination of the protective effect of TNFα on HSPCs and a specific Notch-dependent effect on HTLPs might explain this result.

Therefore, the TNFα-supplemented DL-4 culture system offers a suitable platform for gene correction approaches and establishes new opportunities for developing personalized treatments based on genetically modified T-lymphoid progenitors.

In particular, our culture system could lead to new CAR T-cell approaches based on the transplantation of CAR-modified HTLPs and the generation of naïve and fully functional CAR-modified T cells in patients.

## Supplementary information

Supplemental material

Supplemental material

Supplemetal figures
