# Supplementary material for: A DL-4- and TNFα-based culture system to generate high numbers of nonmodified or genetically modified immunotherapeutic human T-lymphoid progenitors
Source: Cell Mol Immunol. 2021 Jun 11;18(7):1662–76. doi: 10.1038/s41423-021-00706-8 (PMC8245454; doi:10.1038/s41423-021-00706-8)
Supplement: Supplementary file 1 — Supplemental material [file 41423_2021_706_MOESM1_ESM.docx]

**Title:**

**A DL-4- and TNFα-based culture system to generate high numbers of non-modified or genetically modified immunotherapeutic human T-lymphoid progenitors**

**Authors:** Ranjita Devi Moirangthem, Kuiying Ma, Sabrina Lizot, Anne Cordesse, Juliette Olivré, Corinne de Chappedelaine, Akshay Joshi, Agata Cieslak, John Tchen, Nicolas Cagnard, Vahid Asnafi, Antonio Rausell, Laura Simons, Julien Zuber, Tom Taghon, Frank J.T. Staal, Françoise Pflumio, Emmanuelle Six, Marina Cavazzana, Chantal Lagresle-Peyrou, Tayebeh Soheili and Isabelle André*

* To whom correspondence should be addressed: isabelle.andre@inserm.fr

**Supplemental figure legends**

**Fig. S1: Effect of various molecules on cell expansion and differentiation of CD7^+^ HTLPs after 7 days of DL-4 culture of CB or mPB HSPCs.**

**(A, B)** Graphs showing the mean frequencies **(A)** and numbers **(B)** of CD34^+^CD7^+^ progenitors (in black) and CD34^-^CD7^+^ progenitors (in light grey) after 7 days of DL-4 cultures of CB or mPB HSPCs in the presence or absence of 100µM Ph-AA, 750nM SR1, 35nM UM171, 1µM U729 or 100 ng/ml TNFα (mean ± SEM, n=3). The p values were calculated in a One-way ANOVA: **p* ≤ 0.05; ***p* ≤ 0.01; ****p* ≤ 0.001.

**Fig. S2:** **Phenotypic analysis during and after 7 days of DL-4 culture of CB or mPB HSPCs.**

**(A**) The phenotype (from day 3 to day 7) during DL-4 cultures of CB or mPB CD34^+^ HSPCs in the presence (100 ng/ml) or absence of TNFα. **(B)** Analysis of CD7^+^ HTLPs for CD5 and CD1a expression after 7 days of DL-4 cultures of CB or mPB HSPCs in the presence or absence of TNFα.

**Fig. S3: Analysis of TCRδ, γ and β rearrangements in progenitors obtained from DL-4 cultures of CB and mPB HSPCs and HSPC sub-population sorting.**

**(A**) A fluorescent PCR GeneScan® analysis and **(B)** quantification of TCRδ rearrangements among CD7^+^ progenitors generated after 7 days of DL-4 culture of CB or mPB HSPCs under the indicated conditions. Each peak represents the fluorescence intensity of the corresponding rearrangement locus. **(C)** Gating strategy for sorting the HSPC sub-populations.

**Fig. S4: Analyses of the expression of anti-apoptotic proteins in HTLP cultures.**

**(A)** Representative FACS histograms for the expression of anti-apoptotic proteins Bcl-2 and Mcl-1 on day-7 CD7^+^ HTLPs (left panel) and CD7^-^ cells (right panel).

**(B)** The mean frequencies of Bcl-2 and Mcl-1 expressing cells and their corresponding median fluorescence intensities (MFIs) for CD7^+^ HTLPs (left panel) and CD7^-^ cells (right panel) (mean ± SEM, n=3). The p values were calculated using an unpaired two-tailed t test: ****p* ≤ 0.001.

**Fig. S5: TNFRs I and II expression in HTLP cultures.**

**(A)** FACS histograms showing the expression of TNFRs I and II in HSPCs.

**(B, C)** Representative FACS histograms showing the expression of TNFRs I and II in CD7^+^ HTLPs (left panel) and CD7^-^ cells (right panel) **(B)** and their corresponding MFIs **(C)** after 7 days of HTLP cultures of mPB HSPCs in the presence (100 ng/ml) or absence of TNFα or in the presence of TNFα and Piceatannol (25µM) (an NFkB inhibitor) together (mean ± SEM, n=3). The p values were calculated in a one-way ANOVA: **p* ≤ 0.05, ****p* ≤ 0.001.

**Fig. S6: Analyses of the *in vitro* and *in vivo* T-cell potential of CB or mPB HSPC-derived CD7^+^ HTLPs.**

**(A-B)** The representative FACS plots of CD4^+^CD8^+^ cells **(A)** and TCRαβ- or γδ-expressing CD3^+^ cells **(B)** after 1, 2, 3 and 4 weeks of co-culture of day-7 CB or mPB HSPC-derived HTLPs (treated or not with TNFα) with OP9-hDL1 stromal cells.

(**C-E**) A total of 5x10^5^ CD7^+^ CB or mPB HSPC-derived HTLPs (treated or not with TNFα) were intrahepatically transplanted into each neonatal NSG mice. The thymi were analyzed four weeks after transplantation. (**C**) Representative FACS plots showing human CD45^+^ cell engraftment in the thymi. **(D-E)** Representative FACS plots showing the differentiation of CD4^+^CD8^+^ cells **(D)** and TCRαβ- or γδ-expressing CD3^+^ T-cells **(E)** in the thymi.

**Fig. S7: Analyses of the *in vitro* and *in vivo* T-cell potential of CB or mPB HSPC-derived transduced CD7^+^ HTLPs.**

**(A-B)** Representative FACS plots of transduced CD4^+^CD8^+^ cells **(A)** and TCRαβ- or γδ-expressing CD3^+^ cells **(B)** after 1, 2, 3 and 4 weeks of co-culture of day-7 CB or mPB HSPC-derived transduced HTLPs (treated or not with TNFα) with OP9-hDL1 stromal cells.

(**C-F**) A total of 5x10^5^ transduced or non-transduced CD7^+^ CB HSPC-derived HTLPs (treated or not with TNFα) were intrahepatically transplanted into each neonatal NSG mice. The thymuses were analyzed four weeks after transplantation. (**C-D**) Representative FACS plots showing human CD45^+^ **(C)** and transduced hCD45^+^ **(D)** cell engraftment in the thymi. **(E-F)** Representative FACS plots showing differentiation of transduced CD4^+^CD8^+^ cells **(E)** and TCRαβ- or γδ-expressing transduced CD3^+^ T-cells **(F)** in the thymi.
