# Supplementary figures and images for: A DL-4- and TNFα-based culture system to generate high numbers of nonmodified or genetically modified immunotherapeutic human T-lymphoid progenitors
Source: Cell Mol Immunol. 2021 Jun 11;18(7):1662–76. doi: 10.1038/s41423-021-00706-8 (PMC8245454; doi:10.1038/s41423-021-00706-8)

Fig. S1

A

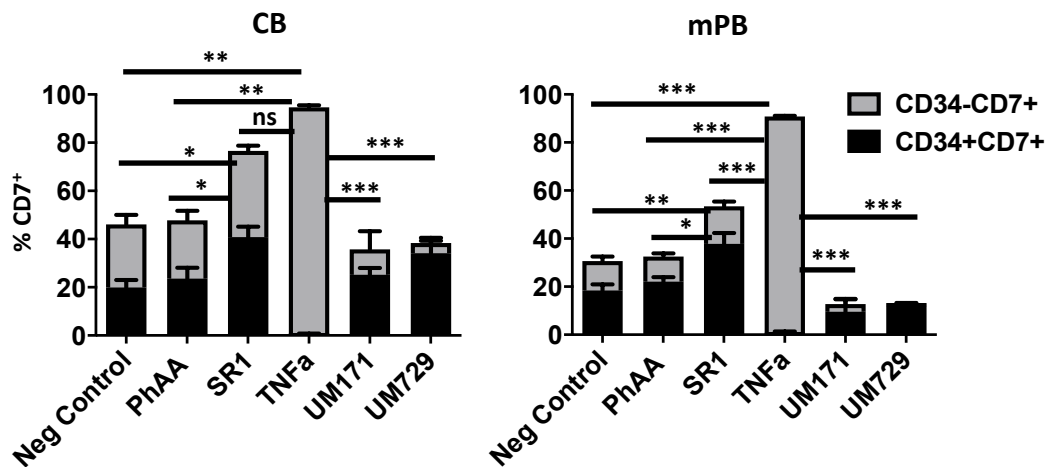

B

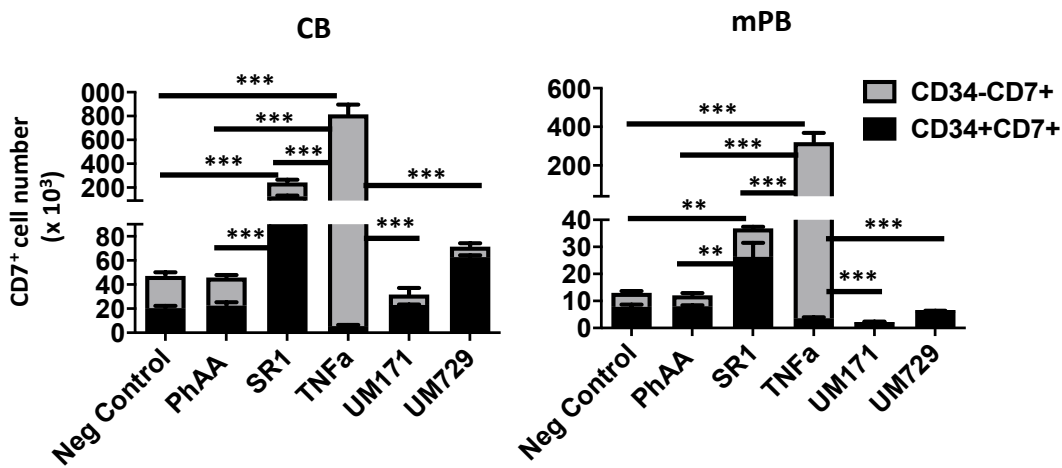

Fig. S2

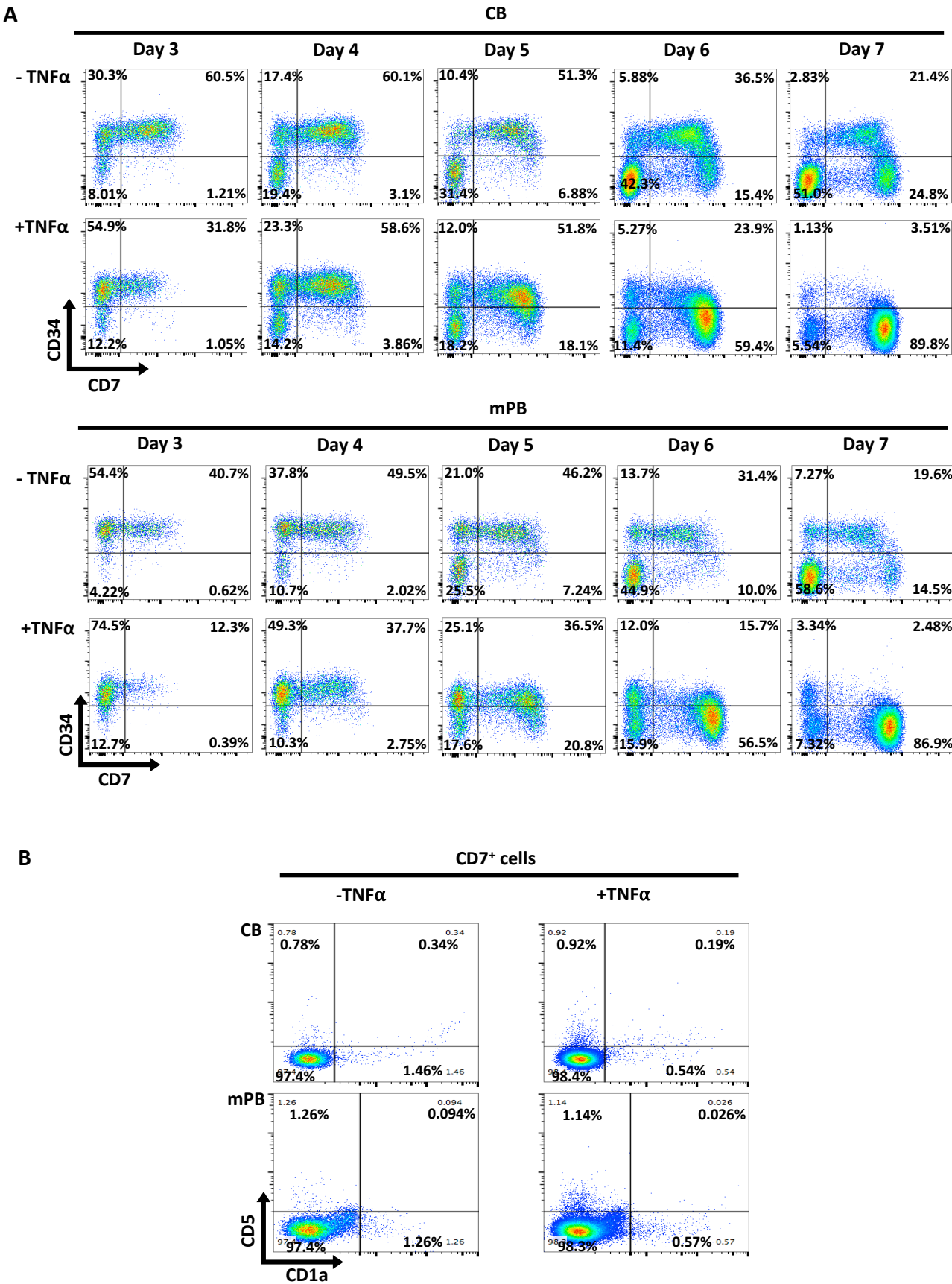

Fig. S3

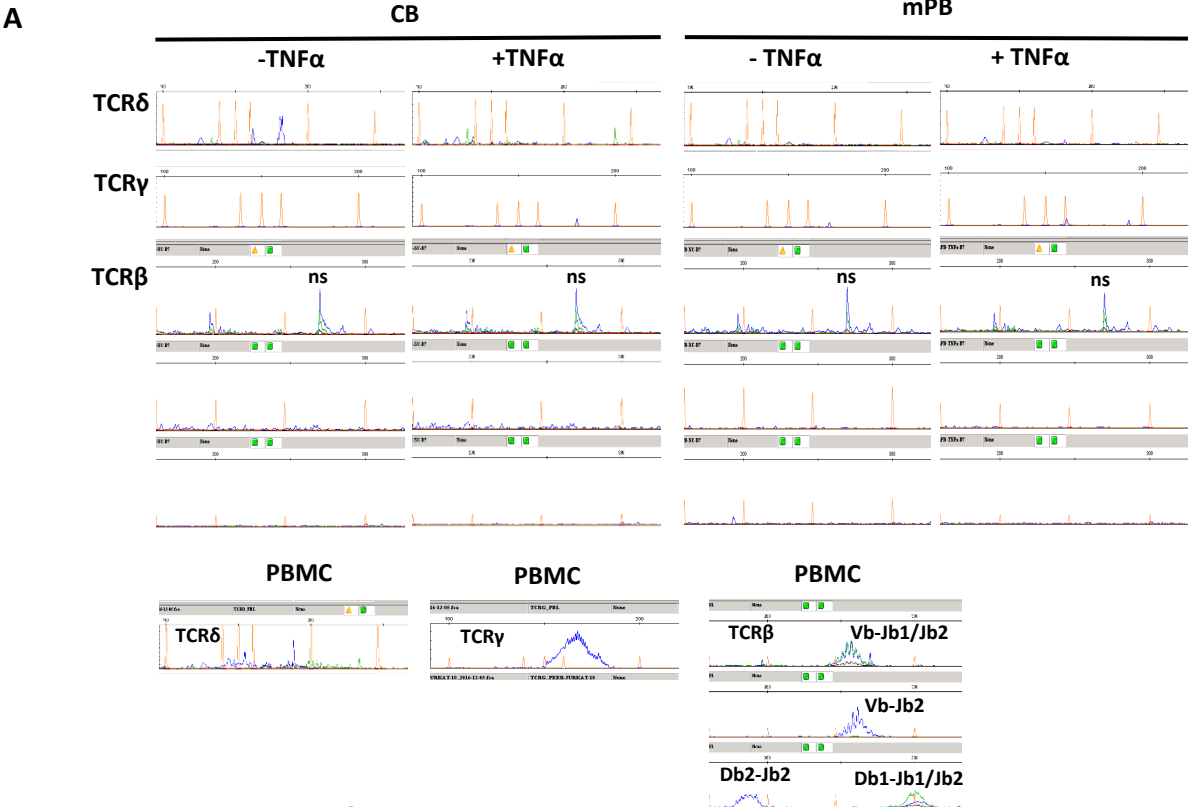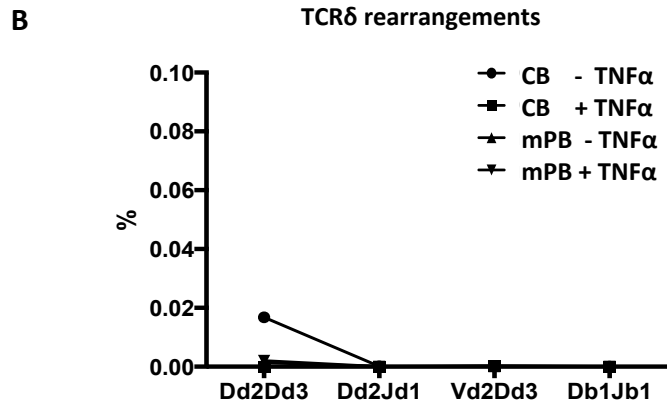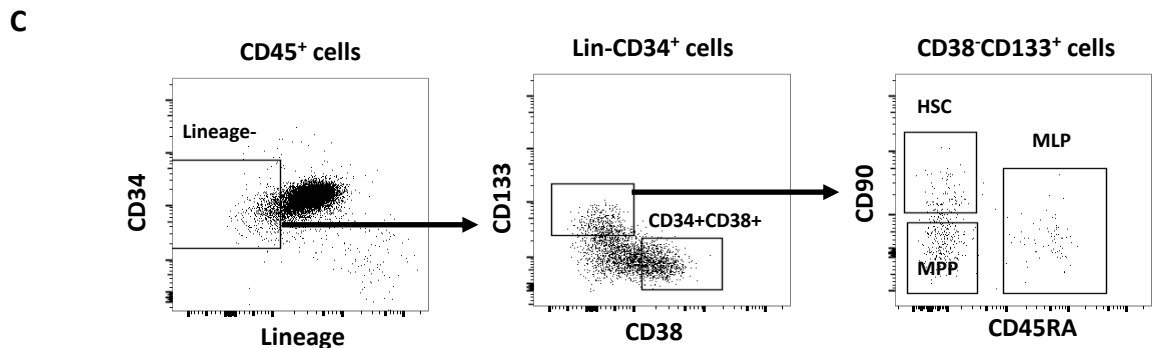

Fig. S4

A

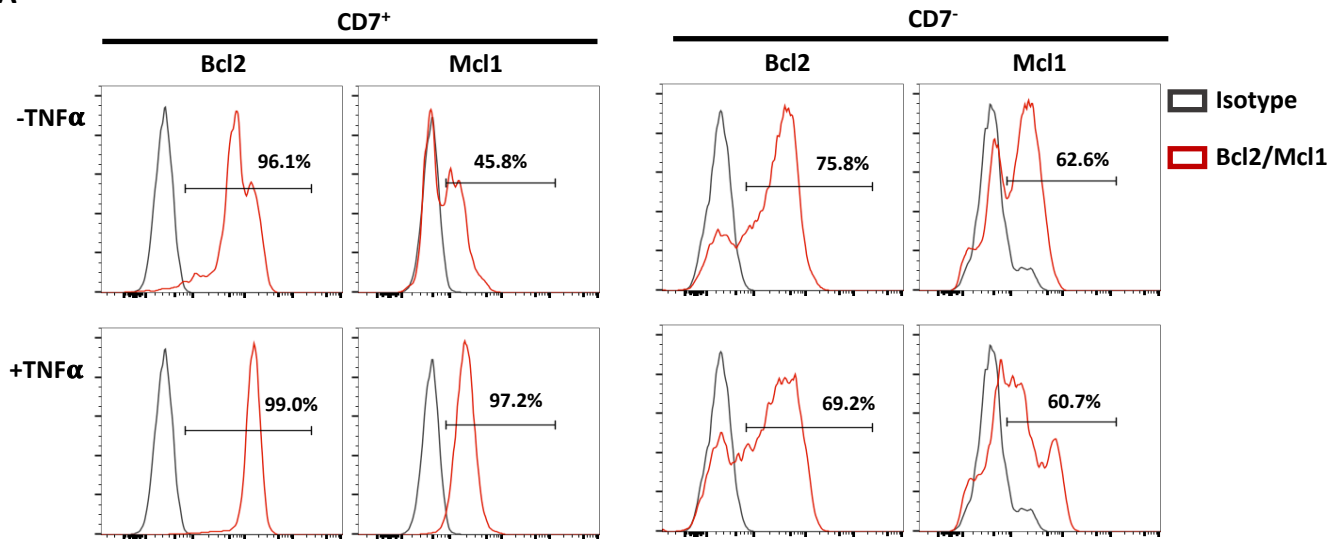

B

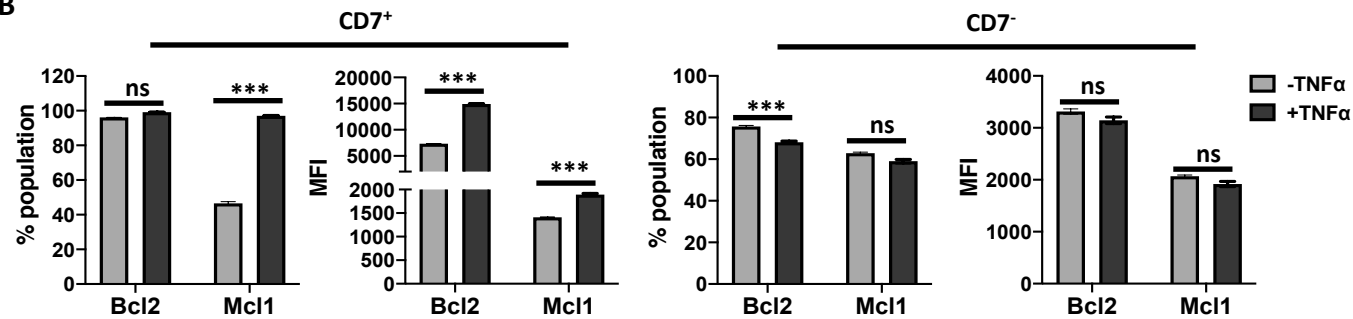

Fig. S5

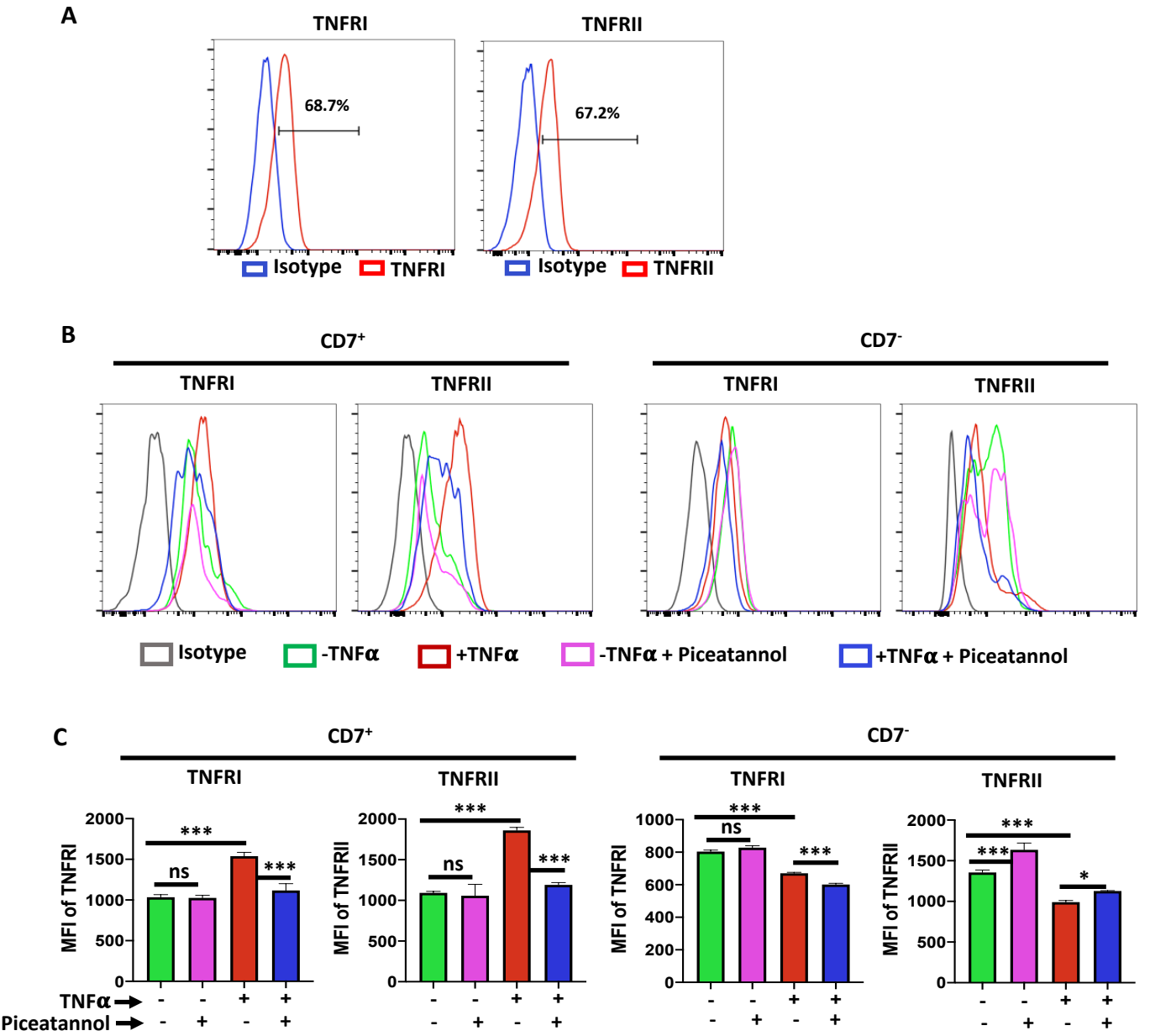

Fig. S6

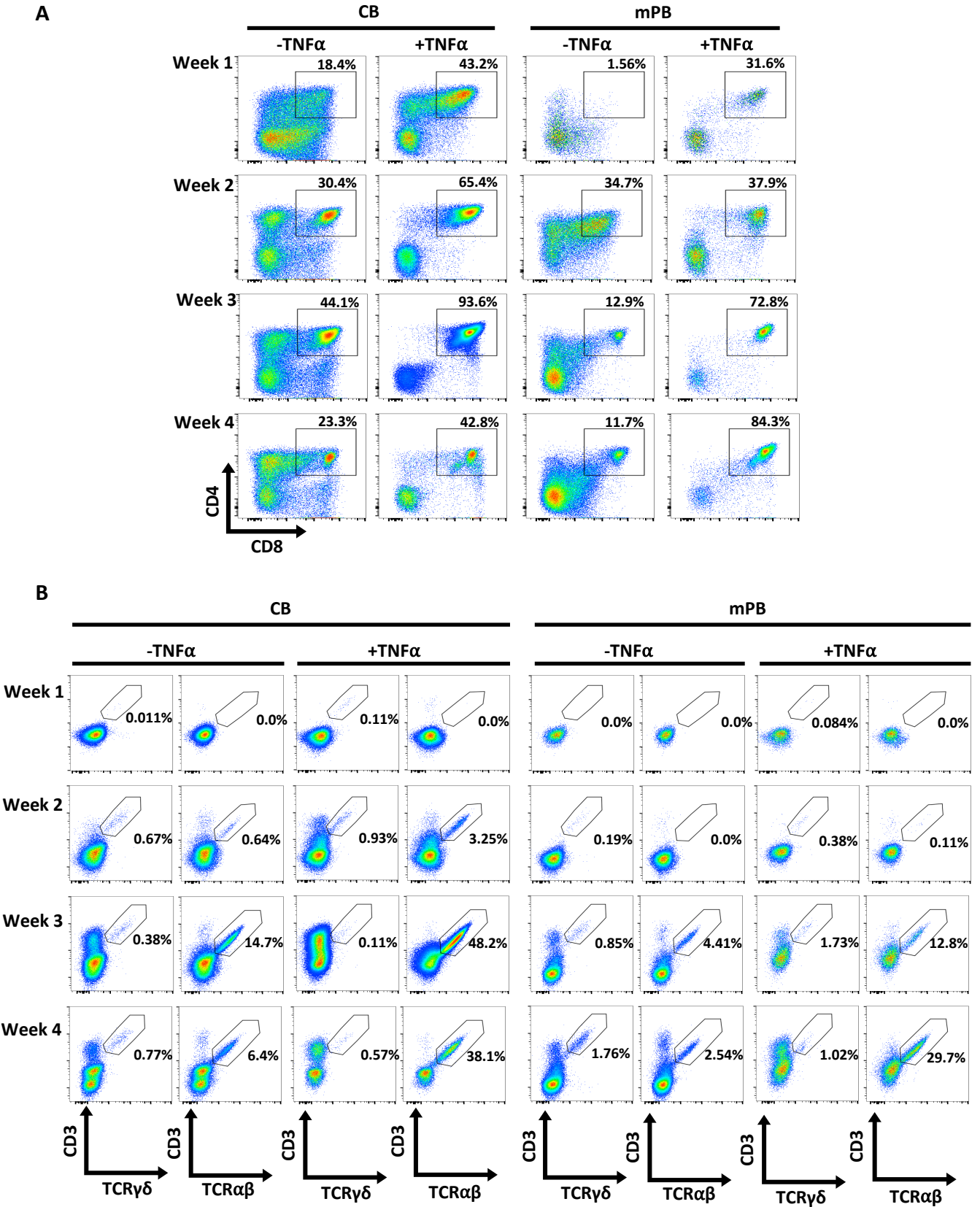

Fig. S6 contd.

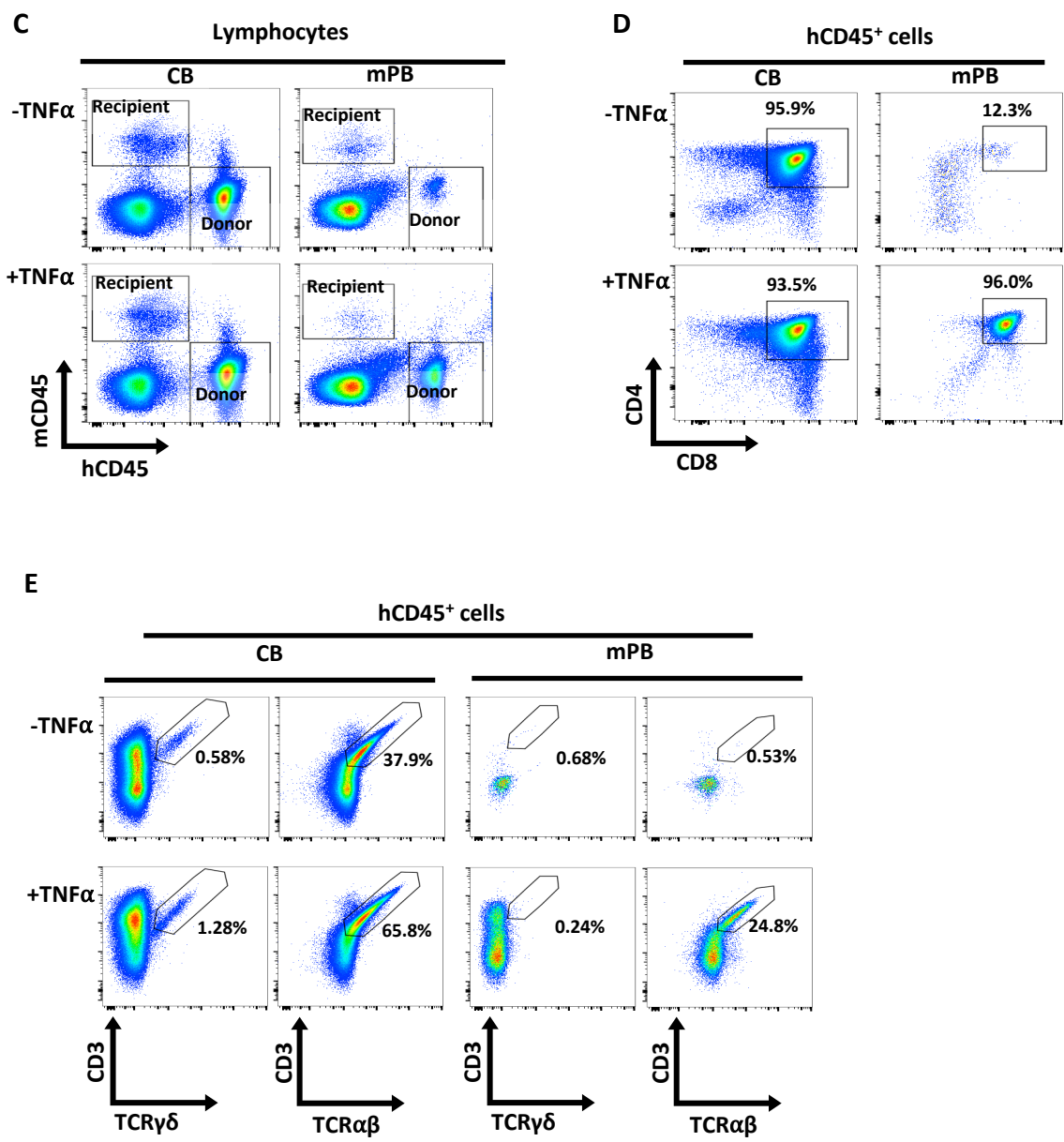

Fig. S7

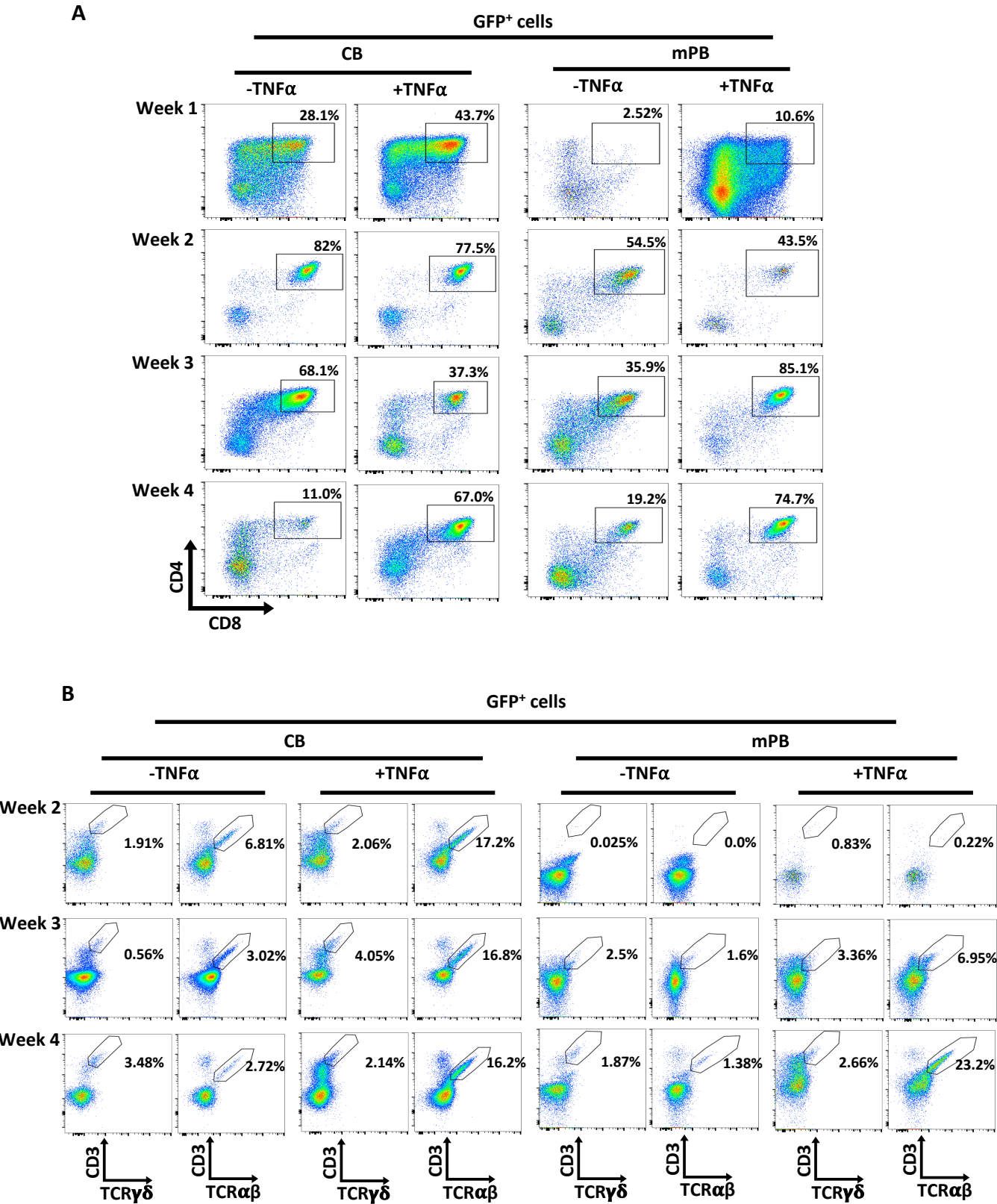

Fig. S7 contd.

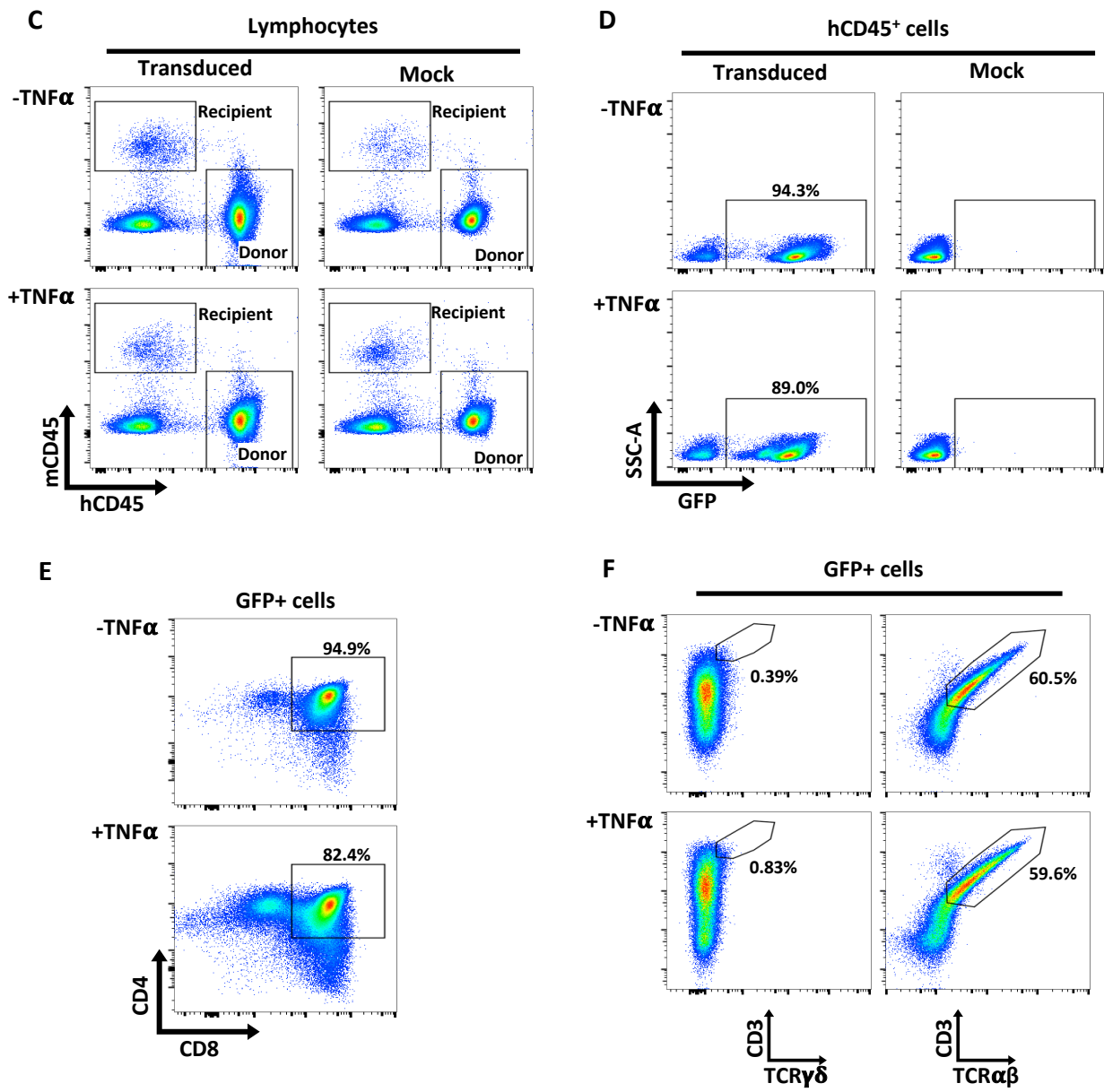

Supplement: Supplementary file 3 — Supplemetal figures [file 41423_2021_706_MOESM3_ESM.pdf]
